# *VHL* loss reprograms the immune landscape to promote an inflammatory myeloid microenvironment in renal tumorigenesis

**DOI:** 10.1172/JCI173934

**Published:** 2024-04-15

**Authors:** Melissa M. Wolf, Matthew Z. Madden, Emily N. Arner, Jackie E. Bader, Xiang Ye, Logan Vlach, Megan L. Tigue, Madelyn D. Landis, Patrick B. Jonker, Zaid Hatem, KayLee K. Steiner, Dakim K. Gaines, Bradley I. Reinfeld, Emma S. Hathaway, Fuxue Xin, M. Noor Tantawy, Scott M. Haake, Eric Jonasch, Alexander Muir, Vivian L. Weiss, Kathryn E. Beckermann, W. Kimryn Rathmell, Jeffrey C. Rathmell

**Affiliations:** 1 Department of Pathology, Microbiology, and Immunology, Vanderbilt University Medical Center, Nashville (VUMC), Tennessee, USA.; 2 Graduate Program in Cancer Biology and; 3 Medical Scientist Training Program, Vanderbilt University, Nashville, Tennessee, USA.; 4 Department of Medicine, VUMC, Nashville, Tennessee, USA.; 5 Ben May Department for Cancer Research, University of Chicago, Chicago, Illinois, USA.; 6 Department of Radiation Oncology,; 7 Vanderbilt-Ingram Cancer Center,; 8 Department of Radiology and Radiological Sciences, and; 9 Vanderbilt University Institute of Imaging Science, VUMC, Nashville, Tennessee, USA.; 10 Department of Genitourinary Medical Oncology, The University of Texas MD Anderson Cancer Center, Houston, Texas, USA.; 11 Vanderbilt Center for Immunobiology, VUMC, Nashville, Tennessee, USA.

**Keywords:** Metabolism, Oncology, Cancer, Macrophages, T cells

## Abstract

Clear cell renal cell carcinoma (ccRCC) is characterized by dysregulated hypoxia signaling and a tumor microenvironment (TME) highly enriched in myeloid and lymphoid cells. Loss of the von Hippel Lindau (*VHL*) gene is a critical early event in ccRCC pathogenesis and promotes stabilization of HIF. Whether *VHL* loss in cancer cells affects immune cells in the TME remains unclear. Using *Vhl* WT and *Vhl*-KO in vivo murine kidney cancer Renca models, we found that *Vhl-*KO tumors were more infiltrated by immune cells. Tumor-associated macrophages (TAMs) from *Vhl-*deficient tumors demonstrated enhanced in vivo glucose consumption, phagocytosis, and inflammatory transcriptional signatures, whereas lymphocytes from *Vhl-*KO tumors showed reduced activation and a lower response to anti–programmed cell death 1 (anti–PD-1) therapy in vivo. The chemokine CX3CL1 was highly expressed in human ccRCC tumors and was associated with *Vhl* deficiency. Deletion of *Cx3cl1* in cancer cells decreased myeloid cell infiltration associated with *Vhl* loss to provide a mechanism by which *Vhl* loss may have contributed to the altered immune landscape. Here, we identify cancer cell–specific genetic features that drove environmental reprogramming and shaped the tumor immune landscape, with therapeutic implications for the treatment of ccRCC.

## Abstract 2

Von Hippel Lindau loss alters immune composition and enhances tumor-associated macrophage function in murine kidney cancer models.

## Graphical abstract

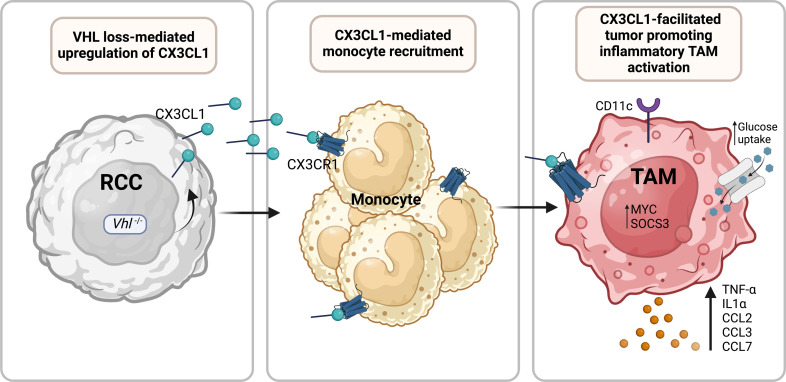

## Introduction

Clear cell renal cell carcinoma (ccRCC) represents the most frequent subtype of RCC, accounting for approximately 75% of adult clinical cases (1, 2). ccRCC biology is unique due to near-ubiquitous loss of function of the von Hippel Lindau (*VHL*) gene, the truncal event in the progression of ccRCC that causes alterations in cellular hypoxia sensing and regulation (2–4). In oxygen-depleted environments or if *VHL* is inactive, HIF proteins are rapidly stabilized and carry out transcriptional upregulation of hypoxia response pathways including angiogenesis and aerobic glycolysis (5). *VHL* loss thus promotes a pseudohypoxic state resulting in characteristic patterns of dysregulated blood vessel formation and may promote shifts in regional nutrient availability and locoregional cellular metabolism (6).

ccRCC tumors have a high frequency of CD8^+^ and CD4^+^ T cells in the tumor microenvironment (TME), making them a favorable candidate for immune checkpoint blockade therapy (ICB) (7, 8). Yet, responsiveness to ICB is variable, and the exact mechanisms that determine responsiveness are not well understood (9–11). Tumor-associated macrophages (TAMs), monocytic and polymorphonuclear myeloid–derived suppressor cells (M-MDSCs and PMN-MDSCs), NK cells, and DCs are other key components of the innate TME that can exert pro- and/or antitumorigenic functions depending on the environment (12, 13). Factors that give rise to lymphoid and myeloid infiltration and fate vary among cancer types for reasons that are poorly understood. In ccRCC, T cells and TAMs are on average the 2 most frequent immune cell populations residing in the TME (13); yet, independent of T cell characteristics, myeloid inflammation has only recently been identified as critical in the development of metastatic ccRCC (14, 15).

Increased glucose uptake in cancer is a common feature used clinically by PET imaging to determine and track disease progression. It is now appreciated that immune and nonimmune cell subsets differ in their capacity to consume nutrients, including glucose, in a tumor setting. Tumor-infiltrating myeloid cells preferentially consume glucose, whereas cancer cells, at least in murine models, have a preference for glutamine uptake (16). However, given the VHL/HIF–mediated axis leading to a robust upregulation in cancer cell aerobic glycolysis, it is possible that *VHL*-deleted ccRCC cancer cells are an exception to this finding so that glucose competition may occur in the TME. Therefore, it is unknown whether *VHL* loss engages reprogramming of the composition of the TME and/or function via metabolic or other functional mechanisms related to constitutive activation of hypoxia response pathways. Appreciation of the effect of *VHL* loss on metabolism and immune infiltration in a tumor setting may provide new opportunities for targeting the TME in ccRCC.

To answer this question, we used the Renca-immunocompetent murine model of kidney cancer, in which we manipulated the *Vhl* axis in vitro to explore the effects on the immune cell repertoire and function in vivo. We found that *Vhl* loss promoted less proliferative tumors and a selective immune microenvironment rich in conventional and regulatory CD4^+^ T cells as well as a distinct subset of TAMs with an increased capacity to consume glucose. *Vhl-*deficient tumors showed minimal response to ICB therapy by day 17, and T cells residing in *Vhl-*deficient tumors expressed fewer markers of dysfunction and effector cytokines when stimulated relative to *Vhl* WT tumors. Interestingly, *Vhl* loss resulted in increased cancer cell production of the cytokine CX3CL1, and deletion of *Cx3cl1*, together with *Vhl*, led to slower tumor growth and decreased myeloid infiltration associated with *Vhl* loss. Together, these findings demonstrate the importance of tumor-specific genetic alterations as a mechanism for reprogramming the immune landscape in cancer and highlight p-VHL loss–mediated CX3CL1 upregulation as a specific driver of myeloid inflammation in ccRCC.

## Results

### Lymphocytes and macrophages are abundant in ccRCC.

**Figure 1:**
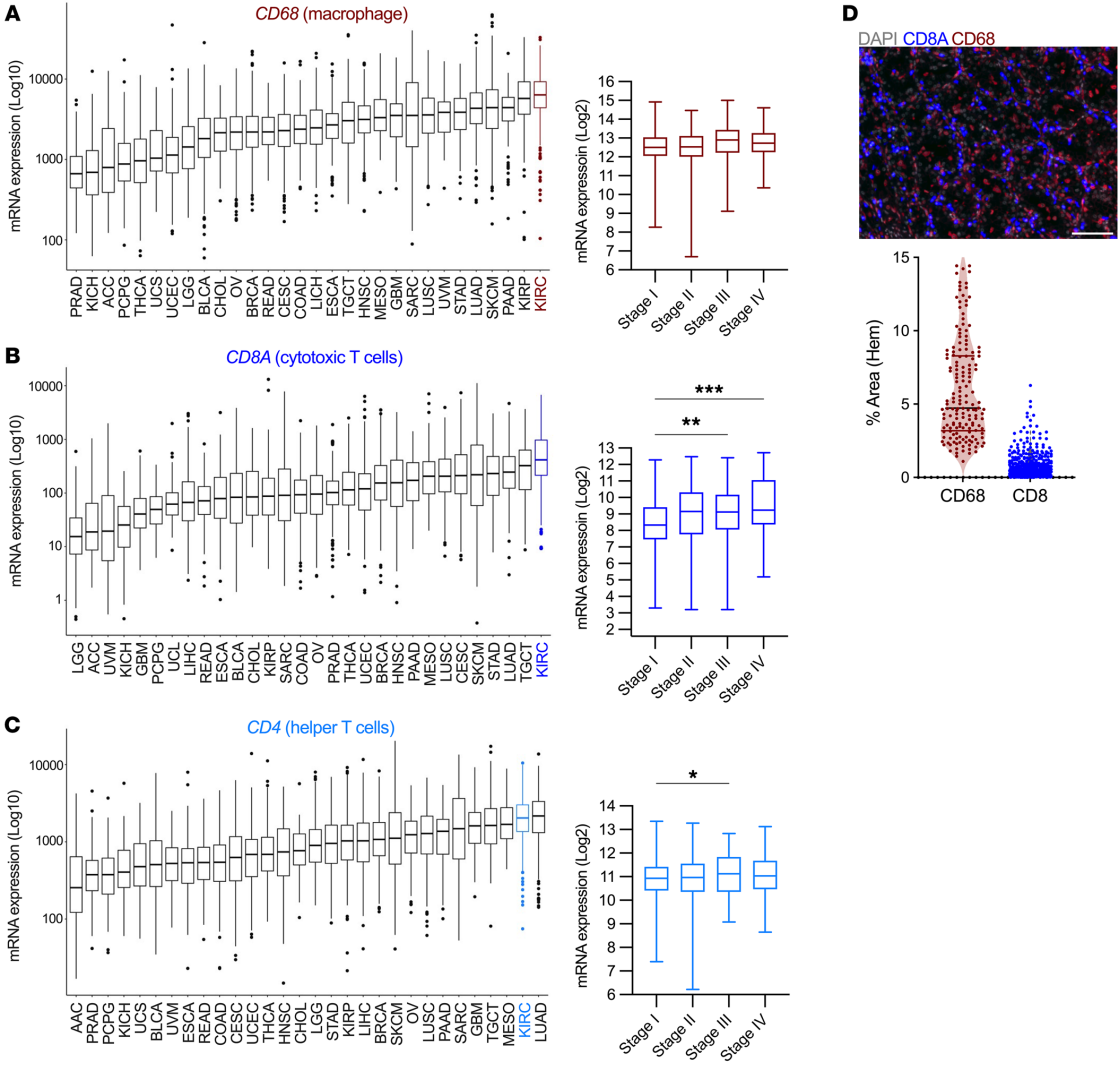
Lymphocytes and macrophages are abundant in ccRCC. (**A**) *CD68*, (**B**) *CD8A*, and (**C**) *CD4* mRNA expression ranked in nonlymphoid solid tumors queried in TCGA. Kidney renal clear cell carcinoma (KIRC) (ccRCC) cells are highlighted with stage-specific expression. *P < 0.05, **P < 0.01, and ***P < 0.001, by Brown-Forsythe and Welch’s ANOVA tests corrected with Games-Howell. (**D**) Representative image of TMA staining identifying CD68^+^ and CD8^+^ cells from patients with ccRCC (scale bar: 50 μm) and quantification of the percentage of area stained with hematoxylin (HEM).

Recent pan-cancer analyses have identified enhanced infiltration of lymphoid and myeloid immune subsets in ccRCC tumors (17, 18). ccRCC exhibits high gene expression of the pan-TAM marker *CD68*, as well as *CD8A* and *CD4* T cell markers from The Cancer Genome Atlas (TCGA) database (Figure 1, A–C). Expression levels of *CD68*, representing all TAMs, and of *CD4* were consistently and highly expressed throughout all stages of tumor progression, whereas *CD8A* expression was increased in later disease stages (Figure 1, A–C). Protein expression observed by high-dimensional multiplex imaging of a human patient tumor among pan-cytokeratin (pan-CK^+^) cancer cells and standard immunofluorescence and quantification by area for CD68 and CD8A on a ccRCC tissue microarray (TMA) staining also indicated diffuse infiltration of CD4^+^, CD8A^+^, and CD68^+^ cells in situ (Figure 1D and Supplemental Figure 1, A and B; supplemental material available online with this article; https://doi.org/10.1172/JCI173934DS1). Additional immune markers associated with *CD14* (monocytes/macrophages), *NCR1* (NK cells), and *CLEC10A* (DCs) were also enriched in ccRCC tumors from TCGA (Supplemental Figure 1, C–E). The exact mechanisms leading to a high immune–infiltrated TME in ccRCC are unknown.

### Vhl loss functionally upregulates HIF targets in the Renca model.

**Figure 2:**
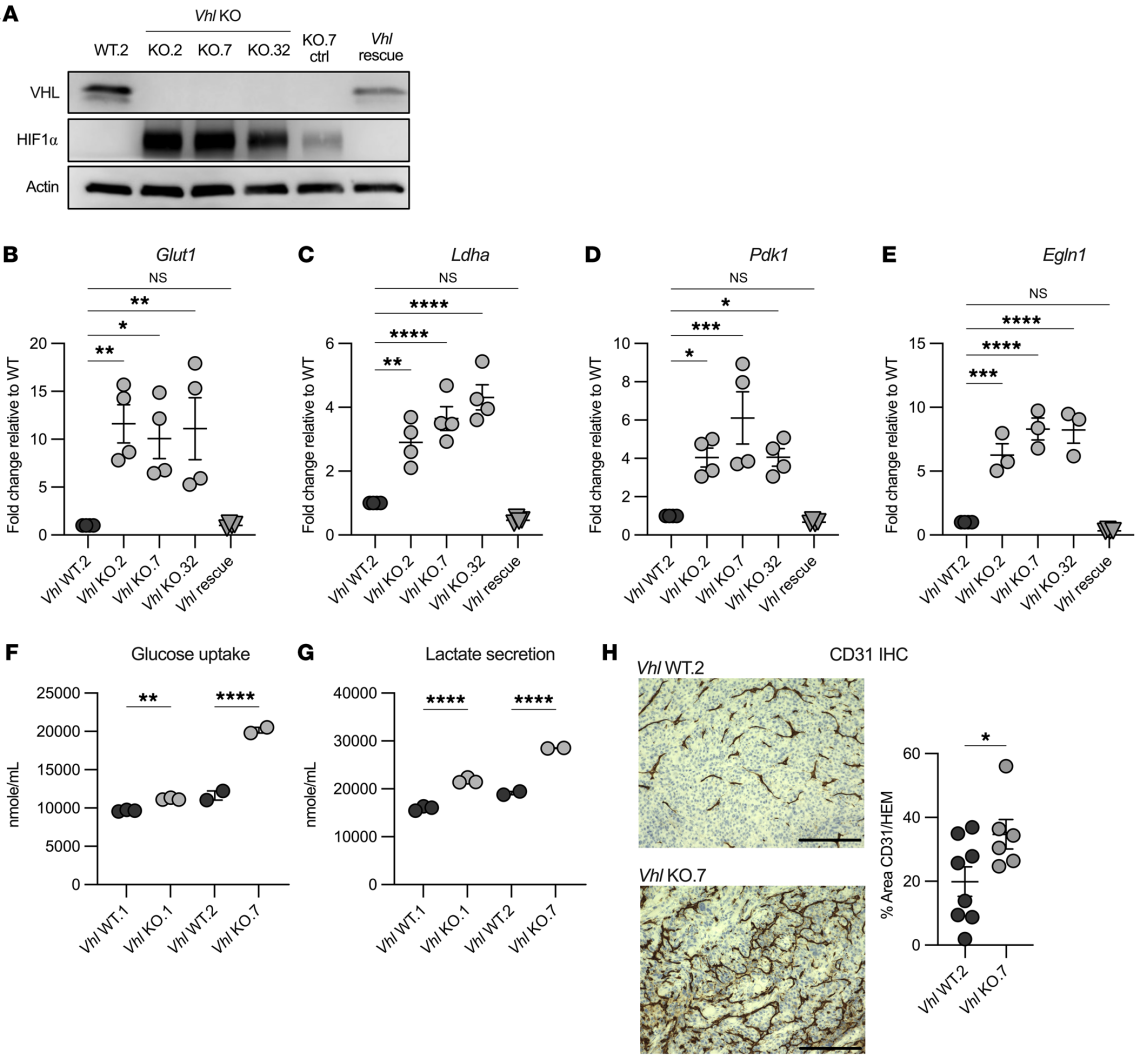
*Vhl* loss functionally upregulates HIF targets in the Renca cell model. (**A**) Representative Western blot showing protein expression of VHL, HIF-1α, and actin in the indicated Renca cell lines. (**B**–**E**) Quantitative PCR of *Glut1*, *Ldha*, *Pdk1*, and *Egln1* in the indicated cell lines relative to *Vhl* WT.2. Each data point represents a technical replicate from 2 independent experiments. Nuclear magnetic resonance (NMR) quantification of glucose uptake (**F**) and lactate secretion (**G**) from CM in Renca *Vhl* WT.1/KO.1 and *Vhl* WT.2/KO.7 paired cell lines. Each data point represents a technical replicate. (**H**) Representative images and quantification of CD31 IHC staining of *Vhl* WT.2 and *Vhl*-KO.7 subcutaneous tumors. Scale bars: 200 μm. *P < 0.05, **P < 0.01, ***P < 0.001, and ****P < 0.0001, by ordinary 1-way ANOVA and Bonferroni’s multiple-comparison test (**B**–**G**) and 2-tailed Student’s *t* test (**H**).

To test whether *Vhl* loss contributes to the increased immune infiltrate observed in ccRCC, we generated clonal *Vhl*-KO Renca murine RCC cell lines using CRISPR editing technology. Renca is a widely used murine RCC line derived from a spontaneous tumor in a BALB/c background and does not contain *Vhl* mutations. To avoid immunogenicity or nonspecific DNA damage from *Cas9* continuous expression, *Cas9* was only transiently expressed. We identified 3 clones from 2 separate guides (KO.2-gA, KO.7-gA, and KO.32-gB) with a marked reduction of p-VHL and increased HIF-1α levels relative to *Vhl* WT (WT.2) (Figure 2A). This phenotype was rescued following lentiviral transduction of either an empty vector control or *Vhl* into KO.7 cells (*Vhl*-KO control/*Vhl* rescue) (Figure 2A). Rescued p-VHL levels were similar but lower than endogenous WT levels, and HIF-1α was no longer detected. We also used a previously characterized *Vhl* WT and -KO Renca pair (*Vhl* WT.1/-KO.1), which demonstrated complete p-VHL loss and increased HIF-1α stabilization (Supplemental Figure 2A) (19).

*Vhl*-KO upregulated HIF targets including *Glut1*, *Ldha*, *Pdk1*, and *Egln1* in all *Vhl*-KO clones at the mRNA level, and reintroduction of *Vhl* successfully rescued mRNA expression of all HIF-1α targets tested (Figure 2, B–E, and Supplemental Figure 2, B–E). We also observed elevated glucose uptake and lactate secretion in vitro with *Vhl* deficiency, consistent with increased aerobic glycolysis (Figure 2, F and G). IHC analysis of subcutaneous tumors formed after injection of *Vhl*-KO Renca cells into syngeneic BALB/c mice demonstrated increased networks of CD31^+^ endothelial cells, confirming enhanced angiogenesis in vivo (Figure 2H). These experiments support the model system as being congruent with the expected biology of *Vhl* loss in a kidney tumor setting. Here, we continued in vivo experimentation using KO.1, KO.7, and KO.32 cells, as *Vhl* was uniquely deleted in each clone, representing biologically independent experiments.

### Vhl deletion slows tumor growth and increases immune infiltration.

**Figure 3:**
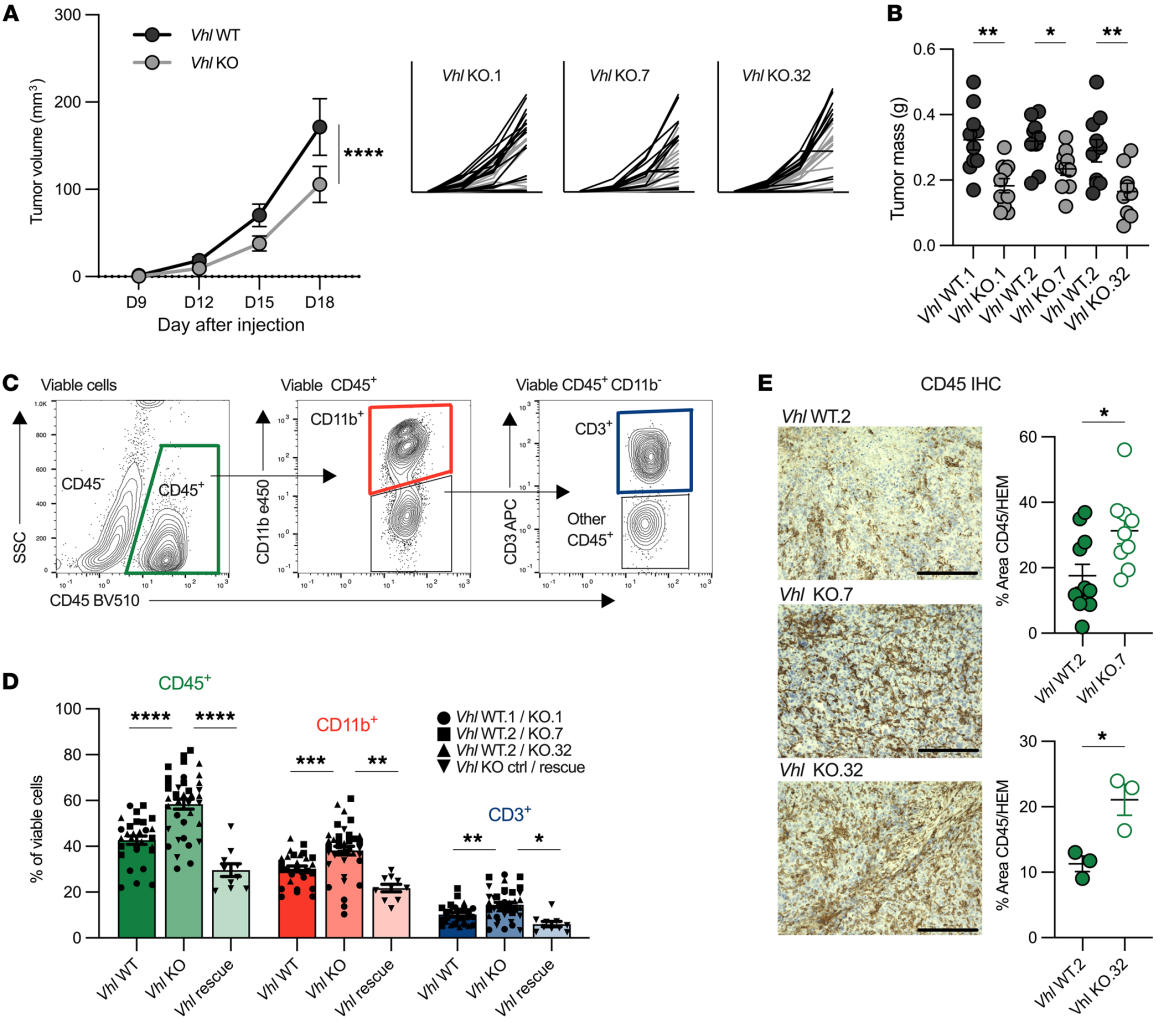
*Vhl* deletion slows tumor growth and increases immune infiltration. (**A**) Average growth curve of all *Vhl* WT (black) and *Vhl*-KO (gray) tumors represented as tumor volume (mm^3^). Smaller graphs represent all biological replicates of paired *Vhl* WT and *Vhl*-KO tumors. *Vhl* WT.1 (*n* = 16) and *Vhl*-KO.1 (*n* = 12); *Vhl* WT.2 (*n* = 14) and *Vhl*-KO.7 (*n* = 14); *Vhl* WT.2 (*n* = 16) and *Vhl*-KO.32 (*n* = 16). D, day. (**B**) Final mass of each tumor. (**C**) Representative gating scheme for CD45^+^, CD11b^+^, and CD3^+^ T cell populations. See Supplemental Figure 3 for complete gating schemes. SSC, side scatter. (**D**) Quantification of CD45^+^ immune cell, CD11b^+^ myeloid cell, and CD3^+^ T cell infiltrate from each *Vhl* WT and *Vhl*-KO pair. WT and KO pairs are represented by matched symbol: *Vhl* WT.1/KO.1 (circle), *Vhl* WT.2/KO.7 (square), *Vhl* WT/KO.32 (triangle), *Vhl* rescue/KO.7 control (reverse triangle). ctrl, control. (**E**) Representative images of CD45^+^ IHC staining in *Vhl* WT.1, *Vhl*-KO.1, and *Vhl*-KO.32 whole tumors. Data are representative of experiments performed at least twice. Graph represents hematoxylin quantification. Each data point represents an individual mouse. Graphs show the mean ± SEM. *P < 0.05, **P < 0.01, ***P < 0.001, and ****P < 0.0001, by 2-way ANOVA and Šidák’s multiple-comparison test (**A**), unpaired, 2-tailed Student’s *t* test (**B** and **E**), and ordinary 1-way ANOVA with Bonferroni’s multiple-comparison test (**D**).

To evaluate the effects of *Vhl* loss on tumor growth dynamics and specific tumor microenvironmental features, Renca *Vhl* WT or *Vhl*-KO cells were grown subcutaneously in BALB/c mice. As previously observed, *Vhl*-KO tumors across clones grew significantly more slowly than did *Vhl* WT tumors (Figure 3, A and B) (20, 21). Considering that this effect may derive from both cell-intrinsic and cell-extrinsic factors, we next hypothesized that the slower-growing *Vhl*-KO tumors with increased angiogenesis might also display altered immune infiltration. Indeed, flow cytometric analyses revealed that *Vhl*-KO tumors harbored an overall increased proportion of CD45^+^ immune cells, including CD11b^+^ myeloid cells and CD3^+^ T cells (Figure 3, C and D, and Supplemental Figure 3A). IHC confirmed the overall increase in CD45^+^ cells in *Vhl*-KO tumors in situ (Figure 3E). We observed enhanced immune infiltration across independent clones (Figure 3D). The increased immune infiltration observed did not correlate with differences in tumor size (Supplemental Figure 2H). To test whether the increased immune cell infiltration was directly influenced by the absence of *Vhl*, we used our *Vhl* rescue clone. Reintroduction of *Vhl* into KO.7 cells did not result in increased tumor volume (Supplemental Figure 2, F and G). However, we observed that CD45^+^, CD11b^+^, and CD3^+^ cell infiltration returned to previous levels compared with the *Vhl*-KO control. These data confirm the specific importance of *Vhl* loss to promote immune infiltration in our models (Figure 3D).

### Increased TAMs displaying proinflammatory properties reside in the Vhl-KO TME.

**Figure 4:**
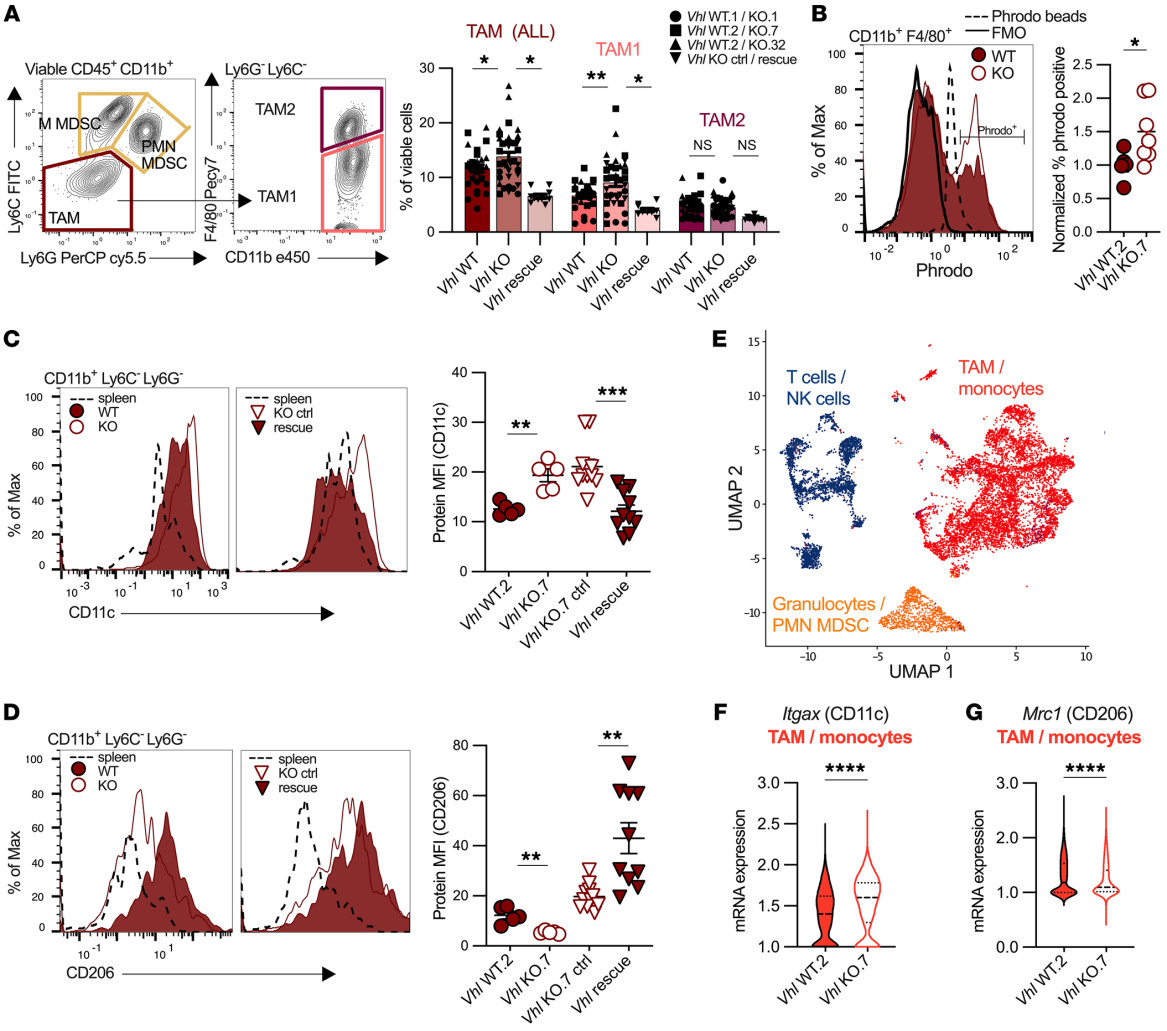
Increased TAM displaying proinflammatory properties reside in the *Vhl-*KO TME. (**A**) Representative flow plots of myeloid cell populations in viable CD45^+^CD11b^+^ cell populations defined as PMN-MDSC (Ly6G^+^Ly6C^lo-int^), M-MDSC (Ly6G^–^Ly6C^hi^), overall TAMs (Ly6G^–^Ly6C^lo^), and the TAM subsets TAM1 (Ly6G^–^Ly6C^lo^F4/80^lo-int^) and TAM2 (Ly6G^–^Ly6C^lo^F4/80^hi^), and quantification of overall TAM, TAM1, and TAM2 infiltration as a percentage of viable cells in each *Vhl* WT and *Vhl*-KO pairs (pairs are represented by a matched symbol). See Supplemental Figure 3 for complete gating schemes. (**B**) Percentage of Phrodo^+^ cell populations as a fraction of viable CD45^+^CD11b^+^F4/80^+^ cells in *Vhl* WT.2 and *Vhl*-KO.7 tumors. Protein MFI quantification and representative histogram of (**C**) CD11c and (**D**) CD206 in overall TAMs from *Vhl* WT.2, *Vhl-*KO.7, *Vhl* rescue, and *Vhl*-KO.7 control tumors. (**E**) UMAP of CD45^+^ scRNA-Seq and mRNA expression levels of (**F**) *Itgax* (CD11c) and (**G**) *Mrc1* (CD206) in TAMs/monocytes from *Vhl* WT.2 or KO.7 tumors. Each data point represents a biological replicate, and graphs show the mean ± SEM. *P < 0.05, **P < 0.01, ***P < 0.001, and ****P < 0.0001, by ordinary 1-way ANOVA with Bonferroni’s multiple-comparison test (**A**), 2-tailed Student’s *t* test (**B**, **C**, and **D**), and Wilcoxon rank-sum test with a threshold of *q* < 0.05 (**F** and **G**). Max, maximum.

We next sought to determine if the CD11b^+^ phenotype observed in the *Vhl*-KO clones consisted of an overall increase in all myeloid cells or if it was limited to specific cell populations. Among innate immune cells, we found that NK cell, DC, and TAM populations were elevated in *Vhl*-KO tumors (Figure 3A and Supplemental Figure 3, A–C). No consistent change was observed in the frequency of infiltrating M-MDSC or PMN-MDSC subtypes (Supplemental Figure 3D). Most notably, the frequency of overall TAM infiltration was markedly increased in the *Vhl*-KO TME (Figure 4, B and C, and Supplemental Figure 3A). Further subdivision of TAM subsets into either TAM1 (CD11b^+^Ly6G^–^Ly6C^lo^F4/80^lo-int^) or TAM2 (CD11b^+^Ly6G^–^Ly6C^lo^F4/80^hi^) revealed that TAM1 frequency was elevated in the *Vhl*-KO TME, while TAM2 infiltration was not changed (Figure 4, A and B). *Vhl* reintroduction rescued the *Vhl*-KO phenotype to restore all myeloid cell populations, with myeloid markers significantly decreased in the *Vhl* addback compared with the *Vhl*-KO control in both TAM1 and TAM2 populations (Figure 4B).

We next hypothesized that TAMs in *Vhl*-KO tumors were not only more frequent but may be functionally distinct from TAMs in *Vhl* WT tumors. Indeed, we found that TAMs from *Vhl*-KO tumors had an increased ability to phagocytose ex vivo, as measured using a pH-sensitive Phrodo phagocytosis assay. We observed an increase in Phrodo^+^ cells in F4/80^+^ TAMs from *Vhl*-KO tumors (Figure 4B and Supplemental Figure 3E). In addition, CD11c expression, an integrin and marker associated with proinflammatory functions when expressed on CD11b^+^ TAMs (22), was increased in TAMs from *Vhl-*KO across all clones tested , whereas CD206 expression, a mannose receptor and tumor-promoting TAM marker (23), was decreased (Figure 4, D and E, and Supplemental Figure 3, E and F). *Vhl* addback rescued this skewing, restoring protein expression of CD11c and CD206 and suggesting that *Vhl* loss promotes increased TAM of an inflammatory type. RNA expression of CD11c was also higher and CD206 was expressed lower in TAM/monocytes from *Vhl* deficient tumors as assessed by frequency of expressing cells and the level of gene expression by single-cell RNA-Seq (scRNA-Seq) (Figure 4, F and G). Overall, these data demonstrate that *Vhl* loss leads to a microenvironment with increased prevalence of a proinflammatory TAM exhibiting increased capacity for phagocytosis.

### Vhl loss promotes proinflammatory TAM transcriptional signatures.

**Figure 5:**
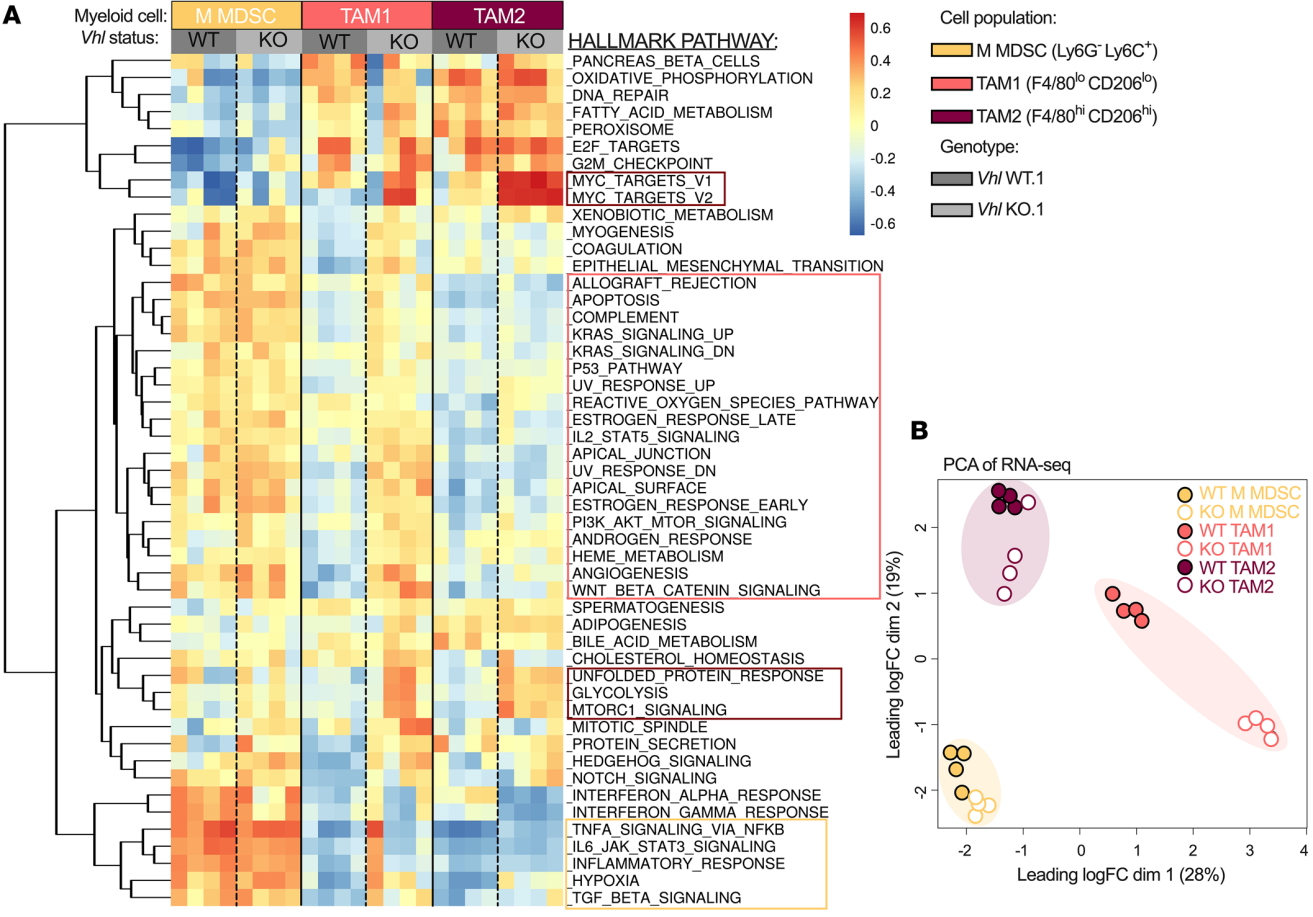
*Vhl* loss promotes proinflammatory TAM transcriptional signatures. (**A**) Heatmap of HALLMARK GSEA scores from flow-sorted cell populations of the indicated cell populations from 4 *Vhl* WT.1 and 4 *Vhl-*KO.1 tumors. Pathways outlined in dark red are enriched in TAM1 and TAM2 from *Vhl-*KO tumors; the pink outline highlights enriched pathways associated with TAM1 from *Vhl-*KO tumors; and the yellow outline highlights pathways enriched in M-MDSCs. (**B**) PCA of RNA-Seq of the indicated cell populations.

To determine whether *Vhl*-KO tumor-infiltrating myeloid cells had a proinflammatory gene signature, we performed bulk RNA-Seq and gene set enrichment analysis (GSEA) on flow-sorted CD11b^+^ cell populations defined as: M-MDSC (Ly6G^–^Ly6C^hi^), TAM1 (F4/80^lo^CD206^lo^), and TAM2 (F4/80^hi^CD206^hi^) (Supplemental Figure 4A). We discovered an enrichment in activation and proinflammatory pathways in both TAM1 and TAM2 from *Vhl*-KO tumors including enriched HALLMARK pathways: MYC targets V1, MYC targets V2, glycolysis, unfolded protein response, and mTOR (Figure 5A). We observed minimal transcriptional changes M-MDSC populations in WT and KO environments. M-MDSC populations from both *Vhl* WT and *Vhl*-KO tumors were enriched for additional inflammatory HALLMARK pathways including the IFN response. Principal component analysis (PCA) revealed clustering according to cell type and *Vhl* status, with TAM1 cells demonstrating a greater degree of separation between WT and KO conditions (Figure 5B).

Additional shifts in individual gene transcripts revealed substantial variations in regulatory transcription factors, surface markers, and secreted factors associated with TAMs from genetically distinct tumors. Expression of the regulatory factors *Socs2*, *Irf3*, and *Nfkb1* was significantly elevated in TAMs from *Vhl* WT tumors, whereas *Myc* and *Socs3* expression levels were elevated in TAMs from a *Vhl*-KO TME (Supplemental Figure 4, B and C). Prominent surface proteins on TAMs from *Vhl* WT tumors included the antiinflammation-associated markers *Cd209a*, *Mrc1*, and *Il10ra* (Supplemental Figure 4D). TAMs from *Vhl*-KO tumors exhibited higher transcriptional reads for *Cd14*, *Tlr2*, and *Trem2* (Supplemental Figure 4E). mRNA levels of *Cd68* were similar in TAMs from both microenvironments (Supplemental Figure 4F). mRNA cpm for secretion factors were also divergent in TAMs from WT and KO tumors. *Ccl6*, *Ccl9*, *Ccl17*, *Ccl22*, and *Il1b* expression levels were elevated in TAMs from *Vhl* WT tumors (Supplemental Figure 4G). In contrast, *Ccl2, Ccl3*, *Ccl4*, *Ccl7*, *Ccl12*, *Tnfa*, and *Il1a* expression levels were elevated in TAMs from the *Vhl*-deficient microenvironment (Supplemental Figure 4H). *Nos2* and *Arg1* levels were also elevated in TAMs from the *Vhl*-KO environment, and transcript reads of *Tgfb1* remained similar in TAMs from both WT and KO tumors (Supplemental Figure 4, I and J). These data further support the idea that cancer cell *Vhl* loss mediates not only the quantity of immune infiltration but also a specific inflammatory response in TAMs and define the cell-intrinsic programs of MDSCs and TAMs in these settings.

### Myeloid cells in the Vhl-KO TME are more glycolytic.

**Figure 6:**
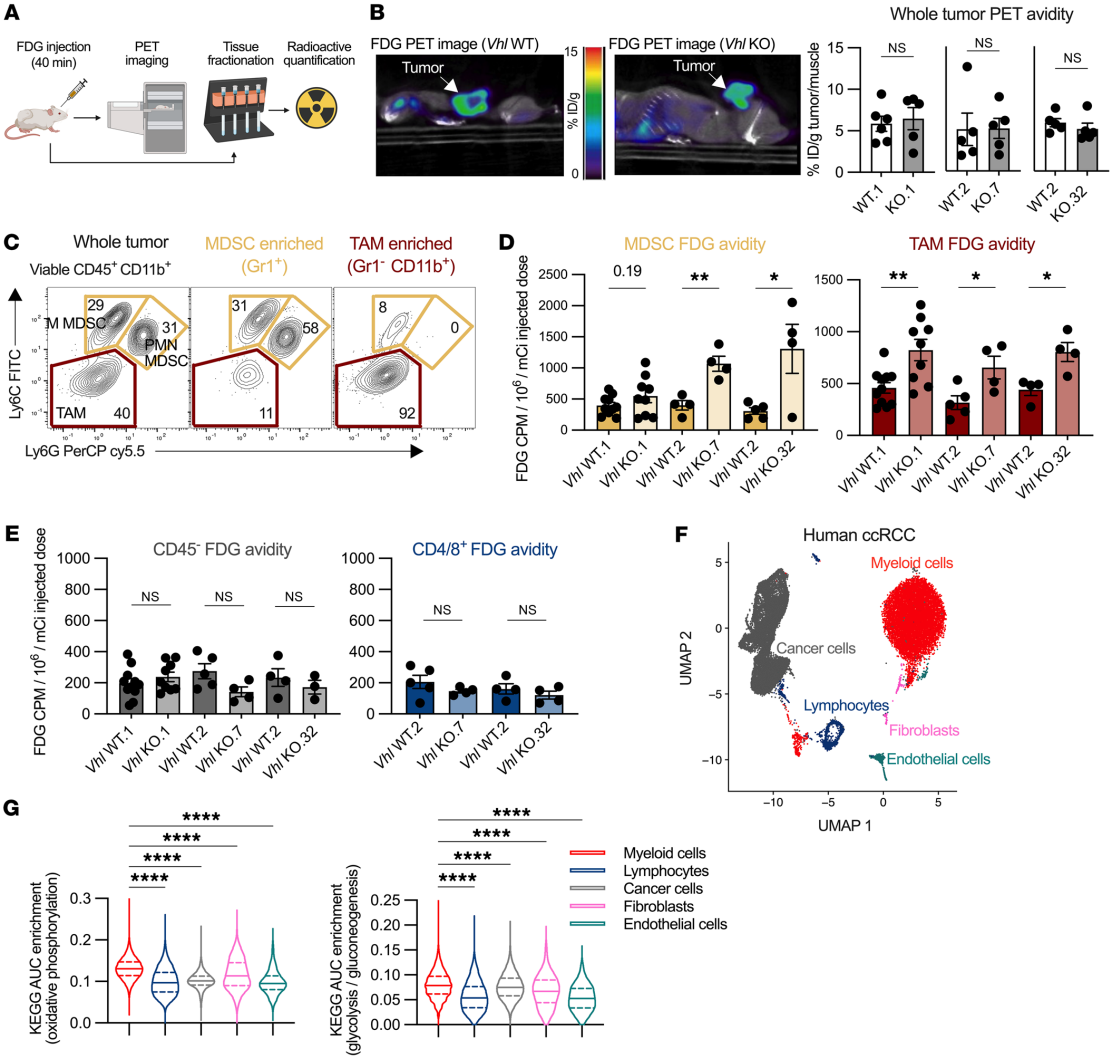
Myeloid cells in the *Vhl-*KO TME are more glycolytic. (**A**) Experimental workflow for FDG PET imaging and characterization of FDG avid single-cell populations. (**B**) Representative FDG PET images of *Vhl* WT and *Vhl*-KO tumors, and quantification of whole tumor PET avidity of the indicated WT and KO pairs. (**C**) Representative flow cytometric gating of whole tumor and MDSC-enriched (anti-Gr1 microbead positive selection) and TAM-enriched (Gr1^–^, anti-CD11b microbead positive selection) populations. (**D**) Quantification of cellular FDG avidity in MDSC-enriched and TAM-enriched cell fractions from the indicated *Vhl* WT and *Vhl-*KO tumors. (**E**) Cellular FDG avidity of CD45^–^ (anti-CD45 microbead negative selection) and CD3^+^ enriched (anti-CD4/CD8 microbead positive selection) T cells from the indicated *Vhl* WT or *Vhl-*KO tumors. (**F**) UMAP showing snRNA-Seq data from 3 human ccRCC patient tumors, and (**G**) KEGG pathway analysis for oxidative phosphorylation and glycolysis/gluconeogenesis in the designated cell populations. Each data point represents a biological replicate, and graphs show the mean ± SEM. *P < 0.05, **P < 0.01, and ****P < 0.0001, by unpaired, 2-tailed Student’s *t* test (**B**, **D**, and **E**) and Brown-Forsythe and Welch’s ANOVAs corrected with Games-Howell (**G**).

In a range of in vivo tumor model settings including Renca tumors, myeloid cells consume more glucose on a per cell basis than do other cell types in the TME (16). However, whether the functional rate of glucose consumption by cells residing in the TME is influenced by genetic factors in cancer cells remains to be explored. Given the p-VHL/HIF axis leading to upregulated glycolytic signatures observed in Figure 2, we first sought to determine whether *Vhl* loss increased functional glucose consumption in *Vhl*-KO tumors relative to *Vhl*-intact tumors in vivo. We performed intravenous injections of the glucose analog tracer [18F]fluorodeoxyglucose (FDG) on tumor-bearing mice followed by either PET imaging or sequential isolation of myeloid cell, T cell, or cancer cell fractions (Figure 6A). Whole tumor PET signals, as well as digested whole tumor fractions and whole spleen fractions, were unchanged in cellular FDG avidity between *Vhl* WT and *Vhl*-KO tumors (Figure 6B and Supplemental Figure 5, A and B). However, enriched fractions of M-MDSCs and PMN-MDSCs (Gr1^+^) and TAMs (Gr1^–^CD11b^+^) from *Vhl*-KO tumors consumed more FDG per cell than did myeloid counterparts from *Vhl* WT tumors (Figure 6, C and D, and Supplemental Figure 5, D–F). *Vhl* status did not alter glucose consumption in isolated tumor-infiltrating CD4^+^ or CD8^+^ T cells or cancer cells enriched as CD45^–^ (Figure 6E and Supplemental Figure 5C). Thus, although *Vhl*-KO cancer cells had increased glycolysis and glucose uptake in vitro (Figure 2, F and G), glucose uptake of *Vhl*-deficient cancer cells was not detectably changed in vivo. Glucose uptake of MDSCs and TAMs in *Vhl*-KO tumors, however, did increase.

To further explore the cell-intrinsic differences in cancer cells, MDSCs, and TAMs in the TME, we conducted scRNA-Seq to compare cells taken from tumors harboring *Vhl* WT or *Vhl*-KO Renca cells. We performed Kyoto Encyclopedia of Genes and Genomes (KEGG) GSEA to determine metabolic pathway enrichment of our clusters. Consistent with their genotype but opposite of their in vivo FDG uptake, cancer cells in the *Vhl*-KO CD45^–^ fraction were significantly enriched for glycolysis and gluconeogenesis transcriptional signatures relative to WT cells (Supplemental Figure 6A). TAMs, monocytes, and MDSCs from the *Vhl*-KO microenvironment were highly enriched in glycolysis, whereas T cells and NK cells showed no change, consistent with our functional in vivo assays (Supplemental Figure 6B). These data suggest that the in vivo functional metabolism was complex in the TME of *Vhl*-deficient tumors, where myeloid cells had the capacity to consume more glucose and cancer cells upregulate glycolytic pathways without functionally consuming more glucose.

To identify potential mechanistic drivers of functional metabolic shifts between the genetically defined cancer cells in MDSC and TAM populations, we examined the interstitial fluid to determine if changes in FDG uptake reflect changes in glucose concentration in the TME (24). Glucose and glutamine concentrations were decreased in tumor interstitial fluid (TIF) compared with plasma, but were unchanged between *Vhl* WT and *Vhl*-KO samples (Supplemental Figure 6, C and D). Similarly, the lactate concentration was elevated in tumor samples as compared with plasma concentrations, but was unchanged with regard to cancer cell genotype (Supplemental Figure 6E).

To test our hypothesis that TAMs are highly metabolic compared with other cell subsets in the TME in human ccRCC samples, we first used single-nucleus RNA-Seq (snRNA-Seq) and examined KEGG enrichment scores for both glycolysis and oxidative phosphorylation in 3 patient samples. As expected, the myeloid cell subsets were the most enriched for both glycolysis and oxidative phosphorylation (Figure 6, F and G). We next used fresh tissue to isolate CD11b^+^ myeloid cells and CD3^+^ T cells from 3 ccRCC patient tumors and performed extracellular flux analyses. We observed a higher oxygen consumption rate (OCR) and extracellular acidification rate (ECAR) in the CD11b^+^ cell fraction compared with T cells, as well as interpatient consistency of these readouts (Supplemental Figure 6, F and G). These data are consistent with our findings in murine Renca tumors.

Loss of *Vhl* in RCC tumors enriched for an inflammatory microenvironment compared with other solid tumors, and myeloid cells were a highly glycolytic subset making up the tumor. Our data demonstrate that, although *Vhl* loss promoted upregulation of glycolytic signatures in Renca cancer cells, in vivo uptake of glucose was not changed at a level that was detectable using whole animal imaging modalities or in isolated cancer cells. However, differences were present in myeloid cells at the cellular level. Myeloid cells were not only enriched in the Renca tumors, but both MDSCs and TAMs demonstrated a greater capacity to consume glucose in a pseudohypoxic microenvironment where glucose was not limited. Taken together, these data suggest that p-VHL–deficient tumors may inherently select for an inflammatory myeloid microenvironment.

### T cells residing in the Vhl-KO TME are dysfunctional.

**Figure 7:**
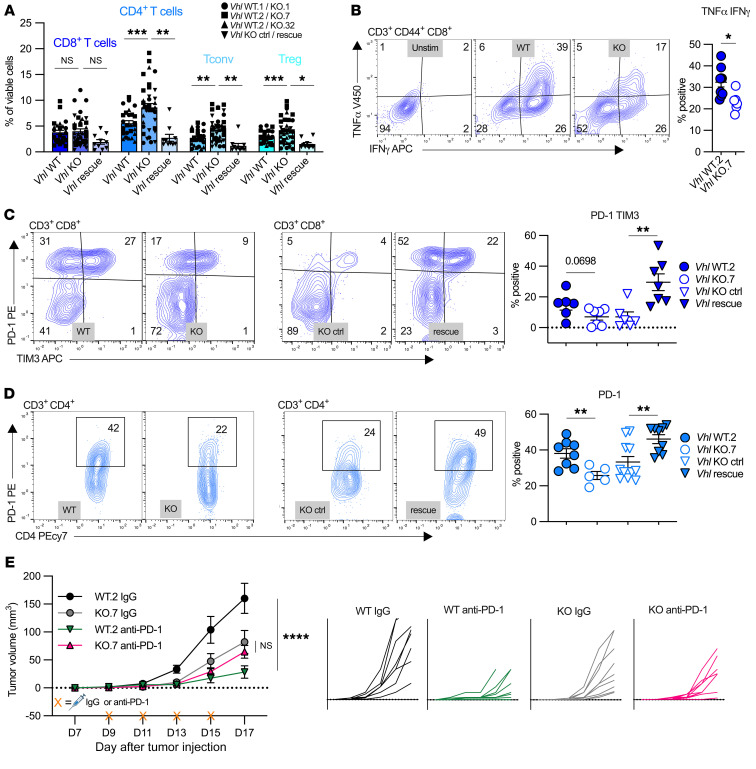
T cells residing in the *Vhl-*KO TME are dysfunctional. (**A**) Quantification of the specified lymphocyte populations as the percentage of viable cells in each *Vhl* WT and *Vhl*-KO pair (pairs are represented by matched symbols). See Supplemental Figure 3 for the complete gating strategy. (**B**) Representative gating and quantification of the percentage of unstimulated and stimulated CD3^+^CD44^+^CD8^+^ T cells expressing TNF-α and IFN-γ in *Vhl* WT.2 or Vhl-KO.7 tumors. (**C**) Representative gating and quantification of the percentage of CD3^+^CD8^+^ T cells expressing PD-1 and TIM3 in *Vhl* WT.2, *Vhl*-KO.7, *Vhl*-KO control, and *Vhl* rescue tumors. (**D**) Representative gating and quantification of the percentage of CD3^+^CD4^+^ T cells expressing PD-1 in *Vhl* WT.2, *Vhl*-KO.7, *Vhl*-KO control, or *Vhl* rescue tumors. (**E**) Volume measurements (mm^3^) over time of IgG- or anti–PD-1–treated *Vhl* WT and *Vhl*-KO tumors. Each data point represents a biological replicate, and graphs show the mean ± SEM. *P < 0.05, **P < 0.01, and ****P < 0.0001, by ordinary 1-way ANOVA with Bonferroni’s multiple-comparison test (**A**), 2-tailed Student’s *t* test (**B**, **C**, and **D**), and 2-way ANOVA with Šidák’s multiple-comparison test (**E**).

Initial immune characterization (Figure 2) revealed an increased abundance of T cells as well as myeloid cells in *Vhl*-KO tumors. Unlike myeloid cells, FDG uptake was unchanged in T cell fractions between WT and KO tumors. To further characterize T cells in our models, we next sought to determine whether T cell subset infiltration, function, and response to immune checkpoint blockade therapy are affected by *Vhl* loss. Our lymphocyte analyses showed that the frequencies of both conventional (Tconv) (FOXP3^–^) and regulatory (Treg) (FOXP3^+^) CD4^+^ T cells were elevated in the *Vhl*-KO TME compared with *Vhl* WT tumors (Figure 7A and Supplemental Figure 3A). CD4^+^ T cell frequencies were increased in tumors of multiple independently derived renal cancer clones and were rescued upon *Vhl* addback (Figure 7A and Supplemental Figure 3A). Notably, the overall frequencies of CD8^+^ T cells and B220^+^ B cells were unchanged by *Vhl* status (Figure 7A and Supplemental Figure 3, A and B).

T cells in *Vhl*-KO tumors were activated, but less so than those in WT tumors. The activation marker CD44 was highly expressed in tumor-infiltrating CD8^+^ and CD4^+^ T cells, but CD8^+^ T cells in KO tumors expressed significantly lower levels of CD44 than did those in WT tumors (Supplemental Figure 7, A and B). The resting marker, CD62L, was similarly downregulated in WT and KO CD8^+^ and CD4^+^ TILs compared with splenic T cells (Supplemental Figure 7, C and D). Following CD4^+^ and CD8^+^ T cell isolation and ex vivo stimulation, CD44^+^CD8^+^ T cells from *Vhl*-KO tumors had a lower capacity to produce the effector cytokines TNF-α and IFN-γ than did WT tumors (Figure 7B). We observed no change in the percentage of TNF-α– or IFN-γ–producing CD44^+^CD4^+^ T cells between WT and KO tumors (Supplemental Figure 7E). We also measured markers related to T cell dysfunction. PD-1– and TIM3-expressing CD8^+^ T cells were less abundant in KO tumors relative to WT tumors (Figure 7C). We found that PD-1 expression in CD4^+^ T cells was also decreased in *Vhl*-KO tumors (Figure 7D). Restoring *Vhl* reversed the presence of TIM3 and PD-1 in both CD8^+^ and CD4^+^ T cells, showing that the *Vhl* status of cancer cells affected T cell function (Figure 7, C and D). We next examined WT *Vhl* and *Vhl*-KO tumor growth in response to anti–PD-1 ICB therapy. Tumor growth measurements revealed that, while *Vhl* WT tumors significantly responded to anti–PD-1 therapy, only a minimal response was observed in *Vhl*-KO tumors on the same treatment schedule (Figure 7E). These data suggest that T cells residing in a pseudohypoxic environment, as a result of *Vhl* loss, have decreased function and response to anti–PD-1 therapy in this model.

### The myeloid CX3CL1/CX3CR1 axis is augmented in Vhl-deficient tumors.

We have identified a cell-extrinsic function related to *Vhl* loss in cancer cells that results in compositional and functional reprogramming in the TME affecting myeloid and lymphoid cells. We next sought to determine the mechanistic underpinnings of this crosstalk. To test whether *Vhl* loss induces specific upregulation of secretory proteins involved in the recruitment and reprogramming of immune cells, we measured a panel of cytokines and growth factors in conditioned media (CM) of *Vhl* WT, *Vhl*-KO, and *Vhl* rescue clones (Supplemental Figure 8A). As expected, protein expression of known HIF targets, including VEGFA and CCL2, was elevated in the *Vhl*-KO supernatant relative to the supernatant of *Vhl* WT and *Vhl* rescue clones (Supplemental Figure 8, B and C). Factors not known to be regulated by HIF, such as GDF-15, were not changed in response to *Vhl* loss (Supplemental Figure 8D). Given the differing growth rates among genetically distinct clones, cytokine abundance was normalized to the cell number. We observed 2 additional chemokines linked to immune recruitment and activation, CX3CL1 (also known as fractalkine) and CXCL16, both of which were elevated in *Vhl-*KO CM compared with WT or rescue CM (Supplemental Figure 8, E and F). CX3CL1 and CXCL16 are chemokines that are known to be upregulated in physiologic hypoxia conditions (25–27) and bind exclusively to their cognate receptors CX3CR1 and CX3CR6, respectively, in mouse and human physiology (28, 29). We analyzed the expression of these receptors in our bulk RNA-Seq data set of flow-sorted myeloid cell populations. *Cx3cr1* was significantly upregulated in PMN-MDSC, M-MDSC, and TAM1 populations residing in *Vhl*-KO tumors and highly expressed in TAM2 in both WT and KO tumors (Supplemental Figure 8E). In contrast, *Cxcr6* expression was negligible in all myeloid cell subsets (<4 cpm) in both WT and KO tumors (Supplemental Figure 8F).

**Figure 8:**
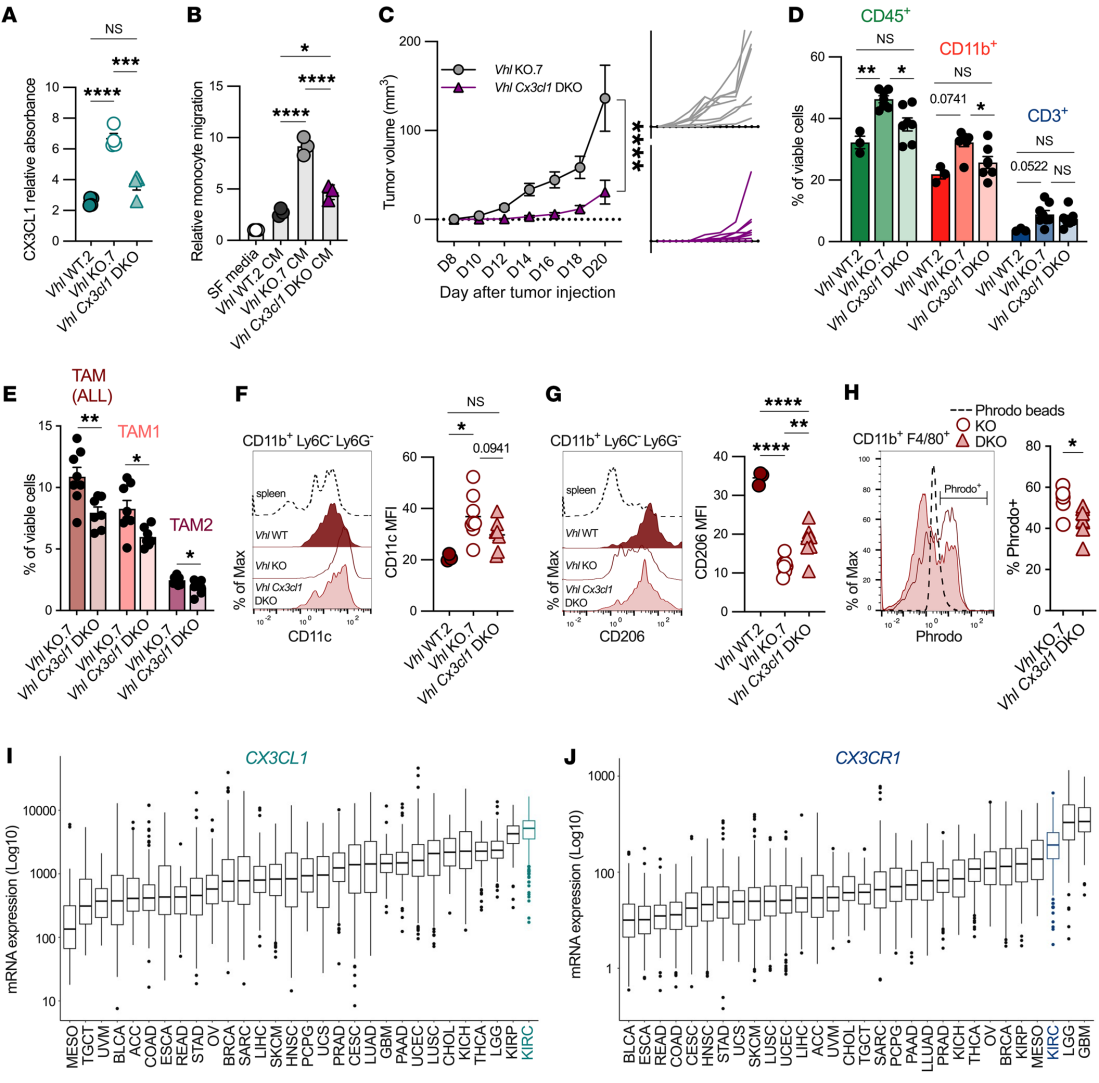
The myeloid CX3CL1/CX3CR1 axis is augmented in *Vhl*-deficient tumors. (**A**) Quantification of soluble CX3CL1 in *Vhl* WT.2, *Vhl*-KO.7, and *Vhl* *CX3CL1*-DKO CM by ELISA. Data represent technical replicates from 2 independent experiments and were normalized per 10^6^ cells. (**B**) Relative monocyte migration stimulated by either SF media or CM from *Vhl* WT.2, *Vhl*-KO.7, or *Vhl* *Cx3cl1*-DKO cells. (**C**) Average growth curve of all *Vhl*-KO.7 (gray) and *Vhl* *Cx3cl1*-DKO (purple) tumors represented as tumor volume (mm^3^). Smaller graphs represent biological replicates. (**D**) Quantification of CD45^+^, CD11b^+^, and CD3^+^ immune infiltrate from *Vhl* WT, *Vhl*-KO, and *Vhl* *Cx3cl1-*DKO tumors. (**E**) Quantification of overall TAM, TAM1, and TAM2 infiltration as the percentage of viable cells in *Vhl*-KO and *Vhl* *Cx3cl1-*DKO tumors (**F**) Protein MFI quantification and representative histogram of CD11c and (**G**) CD206 in overall TAMs from *Vhl* WT.2, *Vhl-*KO.7, and *Vhl* *Cx3cl1-*DKO tumors. (**H**) Percentage of Phrodo^+^ cells as a fraction of viable CD45^+^CD11b^+^F4/80^+^ cells in *Vhl* WT.2 and *Vhl*-KO.7, and *Vhl Cx3cl1-*DKO tumors. (**I**) Ranked gene expression scores for *CX3CL1* and (**J**) C*X3CR1* across 30 nonlymphoid solid tumors queried in TCGA. Data represent biological replicates. *P < 0.05, **P < 0.01, ***P < 0.001, and ****P < 0.0001, by 1-way ANOVA with Bonferroni’s multiple-comparison test (**A**, **B**, **D**, **F**, and **G**), 2-way ANOVA with Šidák’s multiple-comparison test (**C**), and 2-tailed Student’s *t* test (**E** and **H**). Graphs show the mean ± SEM.

To test the potential relevance of CX3CL1 as a mediator of myeloid recruitment and activation in the setting of *Vhl* loss, we deleted *Cx3cl1* from *Vhl*-KO Renca cells to generate a *Vhl Cx3cl1* double-KO (DKO). Quantification of extracellular CX3CL1 by ELISA demonstrated a significant decrease in CX3CL1 in the DKO CM (Figure 8A). We isolated monocytes from the bone marrow (BM) of WT BALB/c mice and performed a Transwell migration assay to test relative monocyte chemotaxis when stimulated by CM acquired from in vitro cultures of *Vhl* WT, *Vhl*-KO, and *Vhl* *Cx3cl1*-DKO clones. *Vhl*-KO supernatant increased monocyte migration relative to *Vhl* WT clones, and this was reversed with DKO CM (Figure 8B). In addition, immortalized BM–derived macrophages (iBMDMs) treated with recombinant mouse CX3CL1 (rmCX3CL1) resulted in increased phagocytosis as well as oxidative metabolism (Supplemental Figure 8, G and H). These data show that CX3CL1 promoted myeloid cell recruitment and shifted myeloid cell function to a proinflammatory state.

The functional characteristics of *Vhl*-KO tumors compared with *Vhl* *Cx3cl1*–DKO tumors were next tested in vivo. DKO tumors grew slower than did *Vhl*-KO tumors (Figure 8C), and overall CD45^+^ and CD11b^+^ infiltration was significantly decreased compared with *Vhl*-KO tumors, to levels comparable to those seen in *Vhl* WT tumors (Figure 8D). TAM populations were also significantly decreased in DKO tumors relative to *Vhl*-KO tumors alone (Figure 8E). In addition, the proinflammatory myeloid cell marker CD11c showed a trend toward a return to the levels seen in *Vhl* WT tumors (Figure 8F). Similarly, the antiinflammation-associated marker CD206 was increased in TAMs from DKO tumors relative to *Vhl*-KO tumors (Figure 8G). A decreased capacity for phagocytosis, as shown by reduced ex vivo Phrodo uptake, was also observed in TAMs from DKO tumors (Figure 8H). CD3^+^ T cell infiltration, T cell subset quantification, and PD-1 expression remained unchanged in the DKO tumors relative to *Vhl*-KO tumors (Figure 8D and Supplemental Figure 8, I–K). CX3CL1 was thus specifically expressed in *Vhl*-deficient tumors and recruited proinflammatory TAMs that may have promoted tumor growth.

To assess the potential relevance of our findings in humans, we performed pan-cancer analyses of *CX3CL1* and *CX3CR1*. We found that *CX3CL1* expression was upregulated in ccRCC tumors and ranked highest in expression among 30 solid tumors (Figure 8I). *CX3CR1* was also highly expressed in ccRCC, ranking third among solid tumors behind myeloid-rich glioblastoma multiforme (GBM) and lower-grade glioma (LGG) (Figure 8J). Overall, these data support the model of *Vhl* deficiency–driven upregulation of the CX3CL1/CX3CR1 axis engagement as a mechanism that leads to increased myeloid infiltration and a distinct tumor-promoting inflammatory TAM state in the TME of RCC.

## Discussion

Here, we describe the cell-extrinsic effects of cancer cell *Vhl* loss in the Renca model of kidney cancer on immune cells residing in the TME. *Vhl* deficiency, which is known to profoundly reprogram the metabolism of cancer cells in vitro, demonstrates additional effects on the composition and function of immune cells in the immediate region. *Vhl* deficiency established a TME rich in CD4^+^ T cells and proinflammatory TAMs that exhibited a unique transcriptional profile, in which regulatory, surface, and secretory factors, as well as functional phagocytosis and glucose consumption, were distinct from TAMs residing in a *Vhl*-intact TME. Although we did not observe an increased abundance of CD8^+^ T cells in the *Vhl*-KO tumors, functionally, they displayed reduced effector cytokine production when stimulated, as well as decreased markers of dysfunction as measured by PD-1 and TIM3 expression. In addition, *Vhl*-KO tumors showed a minimal response to anti–PD-1 treatment compared with *Vhl* WT tumors.

Mechanistically, we observed increased levels of CX3CL1-CX3CR1 in *Vhl-*deficient tumors. Manipulation of this axis by generating a *Vhl* *Cx3cl1*–DKO Renca clone rescued specific myeloid cell characteristics in in vitro and in vivo settings. *Vhl* *Cx3cl1*–DKO conditions promoted slower tumor growth and decreased myeloid cell migration, CD11c expression, and functional phagocytosis. The antiinflammatory marker CD206 was increased in the DKO setting, mimicking the CD206 expression observed in *Vhl* WT tumors. Furthermore, in vitro treatment of rmCX3CL1 on iBMDM cells increased phagocytosis as well as oxidative metabolism. Importantly, DKO tumors showed a significant reduction in growth compared with *Vhl*-KO tumors. These data show that *Vhl* loss mediated CX3CL1 upregulation and played an important role in driving a proinflammatory myeloid cell phenotype. This inflammation may permit or promote tumor growth as well as contribute to T cell dysfunction. These findings demonstrate the importance of tumor-specific genetic alterations to reprogram the immune landscape in cancer and highlight a chronic myeloid cell inflammatory phenotype driven by CX3CL1 as an underlying characteristic and potential target for the treatment of ccRCC tumors.

The chemokine CX3CL1 is known to be regulated by several factors, including hypoxia, although it has never, to our knowledge, been associated with *VHL* loss of function or identified as a mechanism in shaping the TME in ccRCC (28). The CX3CL1/CX3CR1 axis has also been associated with driving a proinflammatory phenotype in disease settings, and administration of an anti-CX3CL1–neutralizing antibody in a model of interstitial lung disease resulted in a decrease of an M1-described inflammatory macrophage infiltration (30).

Until recently, genetic features associated with cancer cells have not been considered to be involved in shaping the functional immune landscape of the TME. *KRAS*-mutant tumors were shown to reprogram macrophages to promote therapeutic resistance and malignant progression by driving the production of CSF2 and lactate in colorectal carcinoma (31). Here, loss of a tumor suppressor promoted changes in macrophage composition and function, skewing toward what appeared to be proinflammatory features. These studies highlight the significance of context-specific cancer cell genetic contributions to the TME in cancer and the influence of tumor cells in modifying the host response.

Secondary to *VHL*, additional mutations (most notably in *PBRM1*, *BAP1*, and *SETD2*) enlist a large number of genetic and epigenetic events to drive advanced tumor evolution (2, 32, 33). While our studies directly tested a role for *VHL*, Renca cells are derived from spontaneously arising murine kidney cancer, in which they were established as *VHL* WT. We were unable, therefore, to fully model the progressive genetic events occurring in human ccRCC that likely affect the TME. Furthermore, some mutations, such as in *Trp53*, are present in both *Vhl* WT and *Vhl*-KO cells at baseline, which may also influence the microenvironment in a way that is challenging to test. Future studies will aim to decipher how additional genetic mutations relevant in ccRCC, including those in *PBRM1*, *SETD2*, and *BAP1*, affect the TME in concert as well as discretely.

Despite an increase in HIF stabilization and glycolytic transcript signatures both in vitro and in vivo, the fractionated *Vhl-*KO CD45^–^ cells isolated from tumors, which predominately consist of cancer cells, did not functionally consume more glucose in our models as compared with *Vhl* WT CD45^–^ fractions. This result contrasts with the functional metabolic findings we observed in vitro and suggests that the metabolic effect of *Vhl* loss in cancer cells may be more complex in vivo, potentially adapting in the setting of the tumor environment and altering metabolic preferences. Shifts in cancer cell metabolic fitness between in vitro and in vivo settings may also be occurring. Future studies will aim to test alternative mechanisms that enrich for glycolysis in *Vhl*-KO conditions without changing the functional consumption of glucose. It is likely that *Vhl*-null and *Vhl-*intact RCC cells utilize glucose in different ways.

Combination ICB therapy (anti–CTLA-4 and anti–PD-1), along with antiangiogenics, is currently the first-line standard of care in metastatic ccRCC (11). However, factors that contribute to patient responsiveness are not well understood, and currently, no reliable biomarker (including PD-1/PD-L1 expression) exists as a predictor of responsiveness to ICB therapy in RCC (34). In our model, *Vhl*-KO tumors showed decreased PD-1 expression and minimal response to anti–PD-1 therapy by day 17 following treatment; however, these data may not comprehensively reflect ICB responsiveness in *Vhl*-KO Renca tumors. For example, given the slower growth rate, future work should evaluate whether *Vhl*-KO tumors exhibit a delayed response to ICB treatment or whether the response is influenced by combination treatment with antiangiogenics.

The observation that T cell abundance and PD-1 expression did not change in *Vhl Cx3cl1*–DKO tumors compared with *Vhl*-KO tumors suggests that *Vhl* loss facilitates multiple mechanisms to affect the microenvironment and the tumor response to ICB. One hypothesis is that CXCL16, a secretory factor identified in our cytokine array assay as being upregulated in *Vhl*-KO tumors (Supplemental Figure 8F), contributes more specifically to T cell dysfunction in the setting of *Vhl* loss, whereas CX3CL1 predominantly alters myeloid cells. In RCC, the expression of CXCL16 and its cognate receptor CXCR6 also associates with better survival outcomes in patients; however, neither has been linked with *VHL* loss or tumor-infiltrating lymphocyte (TIL) infiltration (35). Therefore, future investigations are necessary to determine how the CXCL16/CXCR6 axis or other chemokine pairs may regulate T cell function in the setting of genetically defined RCC tumors.

Proinflammatory macrophages can contribute to tumor-promoting inflammation by secreting proinflammatory cytokines. These molecules can induce immune responses, but they can also support tumor growth and survival of malignant cells. In ccRCC, TAMs may support tumor fitness or disease progression, at least in part, by the proinflammatory action of CX3CL1 driven by *VHL* loss. Thus, *VHL* deficiency in this setting appears to sustain an inflammatory state that affects T cell function and response to therapy.

## Methods

### Sex as a biological variable.

Male and female human ccRCC samples were analyzed. Female mice were used in all murine studies, and it is unknown whether the findings are relevant for male mice.

### TCGA data processing and CODEX fluorescence microscopy.

TCGA data were accessed through the cBioportal and batch normalized from Illumina HiSeq_RNASeqV2 (36). All available cancer types except diseases originating from BM or lymphatic tissue were included. For codetection by indexing (CODEX) staining, FFPE-blocked ccRCC samples were sectioned at 5 μm. Akoya CODEX technology was implemented for spatial imaging. An antibody panel was constructed using the following barcode-conjugated antibody-reporter pairs from Akoya: rabbit anti–CD4-BX003 (clone EPR6855)/Cy5-RX003 (no. 4350018), mouse anti–CD8-BX026 (clone C8/144B)/Atto 550-RX026 (no. 4250012), mouse anti–pan–CK-BX019 (clones AE-1/AE-3)/Alexa Fluor 750–RX019 (no. 4450020), and mouse anti–CD68-BX015 (clone KP1)/Cy5-RX015 (no. 4350019). The CODEX antibodies were conjugated with a unique oligonucleotide sequence.

### TMA staining.

Human ccRCC TMAs were provided by Scott M. Haake (VUMC, Nashville, Tennessee, USA) and the VUMC. Paraffin-embedded TMA slides were prepared for immunofluorescence and stained with anti-CD68 (Cell Signaling Technology, 76437) and anti-CD8 (Cell Signaling Technology, 70306S; 1:500) antibodies as previously described (37). For serial staining, slides were stripped using citric acid buffer, pH 6.1, in a pressure cooker at 110°C for 2 minutes, and then staining was repeated using a different antibody and an Opal fluorophore. After the last Opal staining, the slides were mounted using Antifade Gold Mount with DAPI (Invitrogen, Thermo Fisher Scientific). Stained images were acquired using an Aperio Versa 200 Automated Slide imaging system (Leica/Aperio) via the VUMC Digital Histology Shared Resource (DHSR) core. Images were analyzed with Fiji software. Quantification of markers was done by measuring the total amount of fluorescence divided by the total area of tissue (determined by H&E staining).

### Cell lines.

Renca *Vhl* WT.1 and *Vhl*-KO.1 cell lines were provided by Eric Jonasch (The University of Texas MD Anderson Cancer Center, Houston, Texas, USA) (19). *Vhl* WT.1 was used for CRISPR editing to make additional *Vhl*-KO clones. All Renca cells were grown in RPMI supplemented with 10% FBS, 1% penicillin/streptomycin, 1% l-glutamine, 1% HEPES, 1% sodium pyruvate, and 1% MEM amino acid. For subcutaneous injection, cells were trypsinized and washed 3 times in PBS, and 1 × 10^6^ cells were injected in a volume of 100–200 μL PBS into mouse flanks. Subcutaneous tumors grew for 14–21 days. In immunotherapy studies, tumor-bearing mice received i.p. injections of either 200 μg anti–mouse PD-1 Ab (RMP1-14; Bio X Cell) in 200 μL PBS or 200 μg rat IgG2a isotype control (2A3; Bio X Cell) in 200 μL PBS on days 9, 11, 13, and 15 after tumor injection.

### CRISPR editing.

The following CRISPR guides were used according to the Zhang Laboratory protocol and single-guide scaffold PX458 (38). For *Vhl*-KO.7: VHLCRms1 forward, CACCGCCCGGTGGTAAGATCGGGT; VHLCRms1s reverse, AAACACCCGATCTTACCACCGGGC. For *Vhl-*KO.32: VHLCRms2 forward, CACCGAACTCGCGCGAGCCCTCTC; VHLCRms2 reverse, AAACGAGAGGGCTCGCGCGAGTTC. For *Vhl Cx3cl1–*DKO: CX3CL1CRms1s reverse, CACCGCGGCGCGATGGGAACCTGG; CX3CL1CRms1s, forward AAACACCAGGTTCCCATCGCGCCG. Briefly, 1 μg PX458 was incubated with digest-specific reagents for 30 minutes at 37°C. Digested plasmid was gel purified using the QIAquick Gel Extraction Kit (QIAGEN) and eluted prior to phosphorylation and thermocycler-mediated annealing of each pair of oligonucleotides. Ligation reaction was performed per the Zhang protocol using Quickligation buffer (New England BioLabs [NEB]) and Quick Ligase (NEB) (38).

### Lentiviral transduction.

Vectors were designed and purchased from Vectorbuilder Inc., and HEK293T cells were used to package the vectors into lentiviruses. HEK293T cells were cultured until they reached 60% confluence. For each plate, a transfection media cocktail was prepared using 8 μg of the desired plasmid and a 4:2:1 ratio of PAX2, pMD2.G, and CaCl_2_, respectively. The transfection cocktail was added to the HEK293T cells and incubated for 5–7 hours at 37°C. Transfection media were then discarded and replaced with 10 mL fresh growth media. Media were replaced once more after 8–10 hours, at which point the cells were allowed to produce virus particles for 48–72 hours. The lentiviral supernatants were collected and passed through a 0.45 micron filter and were used immediately.

Lentiviral transduction of Renca cells was performed by mixing 1 mL lentivirus, 6.5 μL of 10 mg/mL polybrene, and 7 mL growth media and incubating for 24 hours at 37°C. The next day, the transduction media were replaced with fresh growth media, and cells were returned to a 37°C incubator for another 24 hours. On the following day, the media of the transduced cells were supplemented with puromycin to select for resistance. The resistant clones were screened for *Vhl*.

### Protein extraction and Western blotting.

Cells were harvested and lysed in RIPA buffer supplemented with 1× Halt protease inhibitor cocktail (Thermo Fisher Scientific) while being kept on ice. Cell lysates were pelleted via centrifugation at 14,000*g* for 5 minutes at 4°C, and supernatants were collected in fresh tubes for immediate SDS-PAGE experimentation or preservation at –80°C. Protein concentrations were determined using BCA protein quantification. For SDS-PAGE, 20–50 μg protein per sample was loaded into and well on a 4%–20% gradient polyacrylamide gel (Bio-Rad), followed by transfer onto a PVDF membrane. The membranes were then subjected to immunoblotting with the primary antibodies VHL (1:100, G7, Santa Cruz Biotechnology, 17780) and HIF-1α (1:1,000, D2U3T, Cell Signaling Technology, 14179), and β-actin (1:1,000, MilliporeSigma, A2066) was used as a loading control.

### 1-H proton nuclear magnetic resonance.

Conditioned culture media were collected at the 24-hour time point after plating 1 × 10^6^ cells in a 6-well plate. Media were processed as previously reported (39). ^1^H-MRS spectra were acquired on an Avance III 600 MHz spectrometer equipped with a Triple Resonance CryoProbe (TCI, Bruker) at 298 K with 7,500 Hz spectral width, 32,768 time domain points, 32 scans (supernatant), and a relaxation delay of 2.7 seconds. The water resonance was suppressed by a gated irradiation centered on the water frequency. The spectra were phased, manually baseline corrected, and referenced to sodium 3-trimethylsilyl-2,2,3,3-tetradeuteropropionate using the Bruker TopSpin-3.6 software package. Spectral assignments were based on literature values.

### Mice.

BALB/c (no. 000651) mice were obtained from the The Jackson Laboratory. Our study exclusively used female mice. It is unknown whether the findings are relevant for male mice. Mice were housed in ventilated cages with, at most, 5 animals per cage and were provided ad libitum access to food and water. Mice were on 12-hour light/12-hour dark cycles, which coincided with daylight in Nashville, Tennessee. The mouse housing facility was maintained at 68°F–76°F and 30%–70% humidity. Eight- to 20-week-old mice were used for injectable tumor models. Mice were euthanized if a humane endpoint was reached (2 cm tumor dimension, ulceration, weight loss >10%).

### IHC.

Mouse tumors were harvested and fixed with 10% neutral buffered formalin solution for 48 hours on a shaker at room temperature. Tissues were then washed in PBS and embedded in paraffin for sectioning by the Translational Pathology Shared Research Core at VUMC. All tissues were sectioned at 5 μm. After sectioning, IHC was performed as previously described (37). Briefly, slides were deparaffinized in xylene and rehydrated in serial ethanol dilutions. Antigen retrieval was performed by heating slides for 17 minutes in Tris EDTA buffer, pH9, in a pressure cooker at 110°C. Slides were cooled and blocked with 2.5% horse serum (Vector Laboratories). After blocking, slides were incubated overnight at 4°C with a primary antibody in horse serum. The following primary antibodies were used: CD31 (1:500, D8V9E, Cell Signaling Technology, 77699) and CD45 (1:500, D3F8Q, Cell Signaling Technology, 70257). Slides were then incubated in anti–rabbit or anti–mouse HRP secondary antibody (Vector Laboratories) for 1 hour at room temperature the following day and subsequently developed using DAB (Vector Laboratories). Image analysis was done using Fiji software as previously described (37).

### RNA isolation and qPCR.

Total RNAs were isolated and purified using the RNeasy Mini Kit (QIAGEN, 74106). Isolated RNA was converted to cDNA using iScript Reverse Transcription Supermix (Bio-Rad). mRNA expression was measured with a real-time PCR detection system (Applied Biosystems) in 96-well optical plates using SsoAdvanced Universal SYBR Green Supermix (Bio-Rad). β-Actin was used as a control.

### Flow cytometry.

Tumor and splenic single-cell suspensions were obtained and incubated in F_c_ block (1:50, BD, 553142) for 10 minutes at room temperature. Cells were washed once with FACS buffer (PBS plus 2% FBS) surface staining was applied for another 15 minutes at room temperature. Cells were then washed again and either resuspended in FACS buffer for data acquisition on Miltenyi MACSQuant Analyzer 10 or 16 or stained for intracellular markers. Ghost Dye Red 780 viability dye (1:4,000, Cell Signaling Technology, 18452S) was treated as a surface marker and used in all staining panels. For intracellular staining, the eBioscience Foxp3/Transcription Factor Staining Buffer kit (Thermo Fisher Scientific, 00-5523-00) was used. Cells were fixed/permeabilized for 20 minutes at 4°C, and then stained for intracellular markers for at least 1 hour at 4°C. The anti-mouse and cross-reactive antibodies used were as follows: CD45 BV510 (1:1,600, 30-F11, BioLegend 103138), B220 e450 (1:400, RA3-6B2, Thermo Fisher Scientific, 48-0452-82), CD11b e450 (1:1,600, M1/70, Thermo Fisher Scientific, 48-0112-82), CD11b FITC (1:1,600, M1/70, BioLegend, 101206), CD8a AF488 (1:1,600, 53-6.7, BioLegend, 100723), Ly6C FITC (1:4,000, HK1.4, BioLegend, 128006), CD11c PE (1:1,000, N418, BioLegend, 117308), FOXP3 PE (1:125, FJK-16s, Thermo Fisher Scientific, 12-5773-82), CD4 PerCP-Cy5.5 (1:600, RM4-5, BioLegend, 100540), Ly6G PerCP-Cy5.5 (1:800, 1A8, BioLegend, 127616), F4/80 PE-Cy7 (1:800, BM8, BioLegend, 123114), NKp46 PE-Cy7 (1:200, 29A1.4, BioLegend, 137618), CD3 APC (1:200, 17A2, BioLegend, 100236), CD206 APC (1:500, C068C2, BioLegend, 141708), FOXP3 PE (1:125, FJK-16s, Thermo Fisher Scientific, 12-5773-82), IFN-γ APC (1:250, XMG1.2, BioLegend, 505810), TNF-α (1:200, MP6-XT22, BD, 560655), PD1 PE (1:150, 29F.1A12, BioLegend, 135214), CD44 PE-Cy7 (1:1,000, 17A2, BioLegend, 1030330), and TIM3 APC (1:100, BioLegend, RMT3-23). For phagocytosis assays, single-cell suspensions were incubated for 45 minutes with 100 mg/mL phrodo red *E. coli* beads (Invitrogen, Thermo Fisher Scientific, P35361). Flow cytometry data were analyzed using FlowJo, version 10.9.0.

### scRNA-Seq sample processing.

Ten-week-old female BALB/c mice were injected subcutaneously with 1 × 10^6^ *Vhl* WT or *Vhl-*KO cells. Tumors were harvested 16 days after injection and then enzymatically digested as described above. CD45^+^ and CD45^–^ live cells were flow sorted and counted using trypan blue and a Bio-Rad TC20 counter. For *Vhl* WT.1 and *Vhl*-KO.1 tumors, 3 tumors of approximately the same weight were pooled together for each treatment group. For *Vhl* WT.2 and *Vhl*-KO.7 samples, CD45^+^ hashing antibodies were applied individually. Flow-sorted cells were incubated with FcX block for 10 minutes at 4°C and then washed with cell staining buffer. Samples were then pooled and resuspended at 500,000 cells/mL in PBS plus 0.4% BSA with a target of 10,000 live cells loaded onto the Chromium Controller (10x Genomics) and processed according to the manufacturer’s instructions. Sequencing was performed on the Illumina NovaSeq 6000, targeting 50,000 reads per cell for the 5′ assay. The raw data (FASTQ files) were demultiplexed and processed using CellRanger (version 6.1.2) to get the gene expression matrix with the reference genome mm10 by VANTAGE.

### snRNA-Seq sample processing.

Nuclei were isolated from patients’ ccRCC tumors as previously described (40). Briefly, tissue cubes were minced and ground using a pestle and Dounce tissue grinder. Homogenate was passed through a strainer, and the tissue flow-through was subjected once again to a pestle and Dounce grinder. Homogenate was then strained, and nuclei were counted using a hemocytometer. Samples were assessed under a microscope to ensure specimens were not contaminated with doublet nuclei in the sample. Samples were resuspended to 10^6^ cells/mL in PBS plus 0.4% BSA, with a target of 20,000 live nuclei loaded onto the Chromium Controller (10x Genomics) and processed according to the manufacturer’s instructions. Sequencing was performed on the Illumina NovaSeq 6000, targeting 50,000 reads per cell for the 5′ assay. The raw data (FASTQ files) were demultiplexed and processed using CellRanger (version 3.1.0) to obtain the gene expression matrix with the reference genome GRCh38 by VANTAGE.

### scRNA-Seq and snRNA-Seq data processing.

Data preprocessing, normalization, integration, and clustering were performed using Seurat (version 4.0.6). In brief, the gene expression matrix for each sample was filtered by keeping cells with more than 200 but fewer than 2,500 detected genes for single nuclei or 8,000 detected genes for single cells, as well as genes that were detected in more than 3 cells. Cells with mitochondrial genes representing greater than 10% (for single nuclei) or 15% (for single cells) of the transcripts were also removed. The matrix for each sample was integrated using a mutual nearest-neighbor algorithm default setting. Clustering was performed via Leiden, and then the cell identity of each cluster was annotated using SingleR. The RNA-Seq read counts for cells were first log normalized with the built-in function “LogNormalized()” provided by the SingleR (version 2.2.0) package with default settings. The cell-type prediction was then done with the normalized matrix using the reference database MouseRNAseqData() provided in the indexing R package celldex (version 1.10.1) and was then manually checked according to the signature gene expression levels of the cell clusters. Differential gene expression analysis was done in Seurat using the function “FindAllMarkers or FindMarkers” with default settings. Data visualization was done using Seurat helper functions.

### Cell-sorting and bulk RNA-Seq analyses.

CD11b^+^ fractions from experimentally matched tumors were isolated with magnetic microbeads prior to staining with viability dye and the indicated surface marker antibodies. The fractions were then sorted on a BD FACSAria III cell sorter and stored in 5 parts RNA cell protect to 1 part FACS solution. RNA was isolated, and low-input RNA-Seq was performed using the SMART-Seq v4 Ultra Low Input RNA Kit. Sample quality control analysis was performed using fluorometry Qubit and integrity by BioAnalyzer. Sample quality was evaluated on the basis of normal ranges provided by the manufacture for (a) binding density (0.1–2.25), (b) field of view (<75), (c) positive control linearity (<0.95), and (d) limit of detection (<2). Samples were excluded if they failed any of the 4 conditions. Two WT-PMN MDSC biological replicates were excluded as outliers for abnormal positive control linearity and limit of detection. Normalization was performed using geometric means based on housing genes, and positive and negative controls. We considered transcripts with an FDR of less than 10% and/or a 2-tailed *t* test *P* value of less than 0.01 as being differentially expressed. All analyses were performed in R (version 4.0.2). The pathway analysis was done with respective gene sets from the molecular signature database with GSVA (version 1.40.1) and clusterProfiler (version 4.8.1) packages.

### PET-CT imaging.

Mouse PET-CT imaging studies were performed as previously described in BALB/c tumor-bearing mice (16). Briefly, mice received a retro-orbital injection of approximately 37 MBq/0.1 mL ^18^F-FDG and were returned to plate-warmed cages. Forty minutes later, the mice were anesthetized under 2% isofluorane and imaged using the Inveon microPET (Siemens Preclinical) for 20 minutes. The raw data was then binned into 3D sinograms with a span of 3 and a ring difference of 47. The images were reconstructed into transaxial slices (128 × 128 × 159) with voxel sizes of 0.0815 × 0.0815 × 0.0796 cm^3^ using the MAP algorithm with 16 subsets, 4 iterations, and a beta of 0.0468. For anatomical coregistration, immediately following the PET scans, the mice underwent a CT scan using NanoSPECT/CT (Mediso) at an x-ray beam intensity of 90 mAs and an x-ray peak voltage of 45 kVp. The CT images were reconstructed into 170 × 170 × 186 voxels at a voxel size of 0.4 × 0.4 × 0.4 mm^3^. The PET-CT images were uploaded into Amide, and volumetric regions of interest were drawn around the tumors. The PET images were normalized to the injected dose, and the mean radiotracer concentration within the ROIs was determined.

### ^18^F-FDG nutrient uptake assay.

BALB/c tumor-bearing mice were retro-orbitally injected with approximately 1 mCi FDG synthesized at VUMC as previously described (16). During radiotracer uptake, the mice were conscious and had access to food and water. Following 40 minutes of incubation with the tracer, mice were euthanized, and the spleen and tumors were collected and prepared into single-cell suspensions. Tumors were mechanically dissociated on the Miltenyi Biotec gentleMACS Octo Dissociator and placed in a 37°C incubator for 30 minutes with digest media containing 435 U/mL deoxyribonuclease I (MilliporeSigma, D5025) and 218 U/mL collagenase (MilliporeSigma, C2674). Tumor cell suspensions were fractionated using serial magnetic bead positive selection according to the manufacturer’s instructions (Miltenyi Biotec kits: CD45 130-110-618, CD4/8 TIL 130-116-480, CD11b 130-049-601, and Gr1 130-094-538). Cell suspensions were applied to LS columns (Miltenyi Biotec, 130-042-401) in Miltenyi QuadroMACS, and fractions were resuspended in 1 mL media. Media (10 μL) were used for trypan blue staining and the TC20 cell count, approximately 50 μL media were stained for flow cytometric determination of fraction cellular composition, and 900 μL media were transferred into 5 mL tubes to measure radioactivity. Splenocyte suspensions (900 μL of 2 mL) and 5 million total cells from the unfractionated whole tumor were also assayed for radioactivity. The Hidex Automatic Gamma Counter was used with 1-minute read times to measure time-normalized ^18^F cpm for each sample. To determine per-cell ^18^F-FDG avidity, the time-normalized cpm was divided by the number of viable cells as determined by the trypan count and normalized to the injected dose (ID) of FDG as measured immediately prior to retro-orbital mouse injection.

### Interstitial fluid collection and gas chromatography/mass spectrometry metabolite analysis.

TIF was collected from freshly harvested *Vhl* WT or *Vhl*-KO1 Renca tumors. Specimens were centrifuged against a 0.22 μm nylon filter (Corning, CLS8169) at 4°C for 5 minutes at 300*g*. Flow-through TIF was flash frozen and stored at –80°C prior to batch analysis. Mouse blood was collected via the submandibular vein, aliquoted immediately into EDTA, and centrifuged for 10 minutes at 850*g* at 4°C. Plasma supernatant was collected and then cleared by centrifugation for 20 minutes at 3,000*g* at 4°C. Gas chromatography/mass spectrometry (GC/MS) quantitation of amino acids was performed as described previously (41). Glucose concentrations in plasma and TIF were measured using a portable glucometer as previously described (42).

### Extracellular flux assay.

Mechanical dissociation of human tumors using Miltenyi gentleMACS (human setting) and enzymatic digestion, as described above, were performed prior to sequential isolations using Miltenyi bead isolation kits (CD11b, 130-049-601 and CD3, 130-097-043). Each isolated fraction was plated at 200,000 live cells/well in 4–8 technical replicates on a Cell-Tak–coated plate (Corning, 354240) in Agilent Seahorse RPMI 1640 supplemented with 10 mM glucose, 1 mM sodium pyruvate, and 2 mM glutamine. Live cells (100,000 cells) were used for iBMDM Seahorse flux assays. Cell metabolism was analyzed on a Seahorse XFe 96 bioanalyzer using the Mitostress assay (Agilent Technologies, 103015-100) with 1μM oligomycin, 2 μM FCCP, and 0.5 μM rotenone/antimycin A. Data were analyzed with Agilent Wave software, version 2.6.

### Cytokine detection assay and ELISA.

Renca cells (10^6^ cells) were plated in complete media containing 10% serum for 24 hours, at which point the media were replaced with complete media containing 2.5% serum for an additional 24 hours. CM were then collected and applied to the Mouse XL Cytokine Array (catalog ARY028) per the manufacturer’s protocol. Image analyses was performed using Fiji software.

The Mouse/CX3CL1/Fractalkine Quantikine ELISA Kit (catalog MCS310) was used on the indicated cell lines for quantification of CX3CL1. CM (1 mL) were collected and dried and then resuspended in 100 μL PBS. Samples were then applied to the ELISA kit per the manufacturer’s protocol.

### Transwell assay.

BM monocytes were isolated from 3 WT BALB/c mice by depletion of nontarget cells using the Monocyte Isolation Kit (BM) (Miltenyi Biotec, 130-100-629). Serum-free (SF) complete medium (600 μL, described above) or SF CM from genetically distinct clones (*Vhl* WT, *Vhl*-KO, or *Vhl Cx3cl1*-DKO) were added to a 24-well plate containing Transwell inserts (Costar 6.5 mm Transwell Permeable Support with 5.0 μm Pore Polycarbonate [PC] Membrane, catalog 3421). Monocytes (100,000 cells) were added to each Transwell insert and incubated at 37°C for 4 hours to allow time for their migration across the membrane. The inserts were carefully removed, and a CellTiter-Glo 2.0 Cell Viability Assay (Promega, G9423) was performed according to the manufacturer’s instructions to detect and quantify viable cells in each well.

### Statistics.

GraphPad Prism 10 (GraphPad Software) was used to create graphs and conduct statistical analyses. Data are expressed as the mean ± SEM. Analyses of the differences between 2 test groups were performed using a nonparametric, unpaired, 2-way *t* test. For analysis of 3 or more groups, a 1-way ANOVA was performed with Dunnett’s or Bonferroni’s test. For the scRNA-Seq violin plot comparing 2 groups, a Wilcoxon signed-rank test was applied. For scRNA-Seq and snRNA-Seq comparing 3 or more groups, a Brown-Forsythe and Welch’s ANOVA were used and corrected with Games-Howell. *P* values were considered statistically significant if *P* was less than 0.05.

### Study approval.

Fresh, histology-confirmed ccRCC tumors were surgically removed from 7 patients included in this study. Supplemental Table 1 contains relevant patient and tumor information. All studies were conducted in accordance with Declaration of Helsinki principles under a protocol approved by the VUMC IRB (protocol 151549). Informed consent was received from all patients prior to inclusion in the study by the Cooperative Human Tissue Network at VUMC. All mouse procedures were implemented under VUMC IACUC-approved protocols and conformed to all relevant regulatory standards.

### Data availability.

All data (see the Supporting Data Values file), including the sequencing data included in this study, are publicly available. The bulk RNA-Seq, scRNA-Seq, and snRNA-Seq have been deposited in the Gene Expression Omnibus (GEO) database (GSE239889).

## Author contributions

MMW, WKR, and JCR conceived and designed the study and composed the manuscript with contributions from the other authors. MMW, MZM, ENA, JEB, MLT, MDL, PBJ, ZH, KKS, BIR, and ESH, performed experiments or assisted with assays. MMW analyzed the data and performed statistical analysis. KEB and SMH provided clinical expertise and human ccRCC samples for snRNA-Seq, extracellular flux assays, and TMA staining. ENA performed TMA and immunohistochemical staining, and DKG performed CODEX staining. LV analyzed TCGA-Seq data, and XY analyzed bulk RNA-Seq, scRNA-Seq, and snRNA-Seq data sets. PBJ and AM performed and analyzed GC/MS of TIF samples. EJ provided materials, and FX and MNT injected and handled mice for ^18^F nutrient uptake assays and performed PET imaging. WKR and JCR obtained funding for this study. VLW provided expertise for monocyte migration assays.

## Supplementary Material

Supplemental data

Unedited blot and gel images

Supplemental table 1

Supporting data values

## Version 1 04/15/2024

Electronic publication
