# Supplementary material for: VHL loss reprograms the immune landscape to promote an inflammatory myeloid microenvironment in renal tumorigenesis
Source: J Clin Invest. 2024 Apr 15;134(8):e173934. doi: 10.1172/JCI173934 (PMC11014672; doi:10.1172/JCI173934)
Supplement: Supplemental data [file jci-134-173934-s136.pdf]

Supplemental data

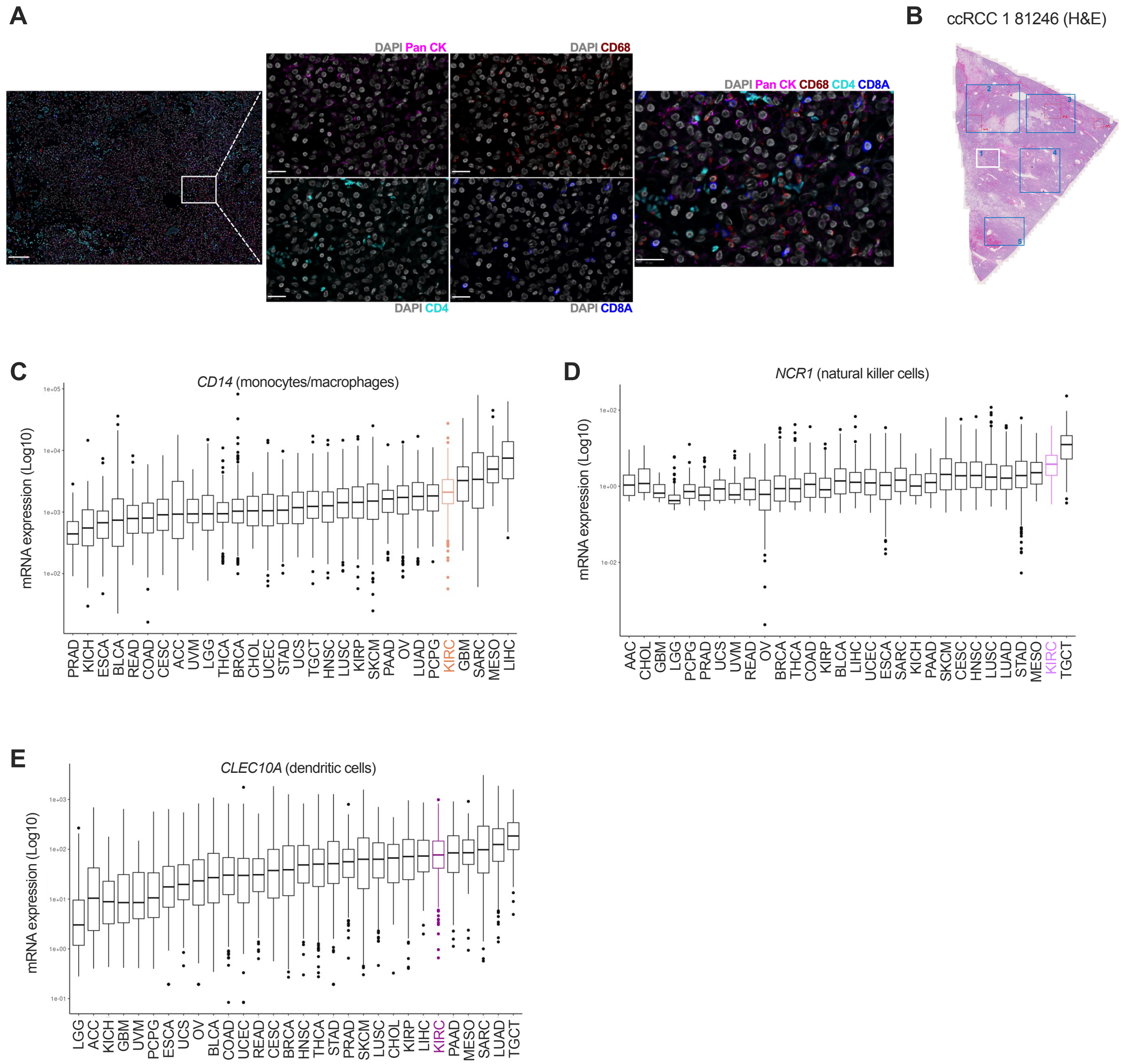

Supplementary Figure 1. Monocytes, natural killer cells, and dendritic cells are abundant in ccRCC.

(**A**) CODEX images representing DAPI (grey), Pan-cytokeratin (Pan-CK) (magenta), CD4 (light blue), CD8A (royal blue), and CD68 (dark red). Scale bar represents 100 □m in zoomed out images; 20 □m in zoomed in images. (**B**) H&E stained ccRCC tumor section regions examined for CODEX analysis in Figure (**A**). Section containing white

box indicates imaged region in A. (**C**) *CD14,* (**D**) *NCR1,* and (**E**) *CLEC10A* mRNA expression in non-lymphoid solid tumors queried in TCGA. KIRC (ccRCC) tumors are highlighted.

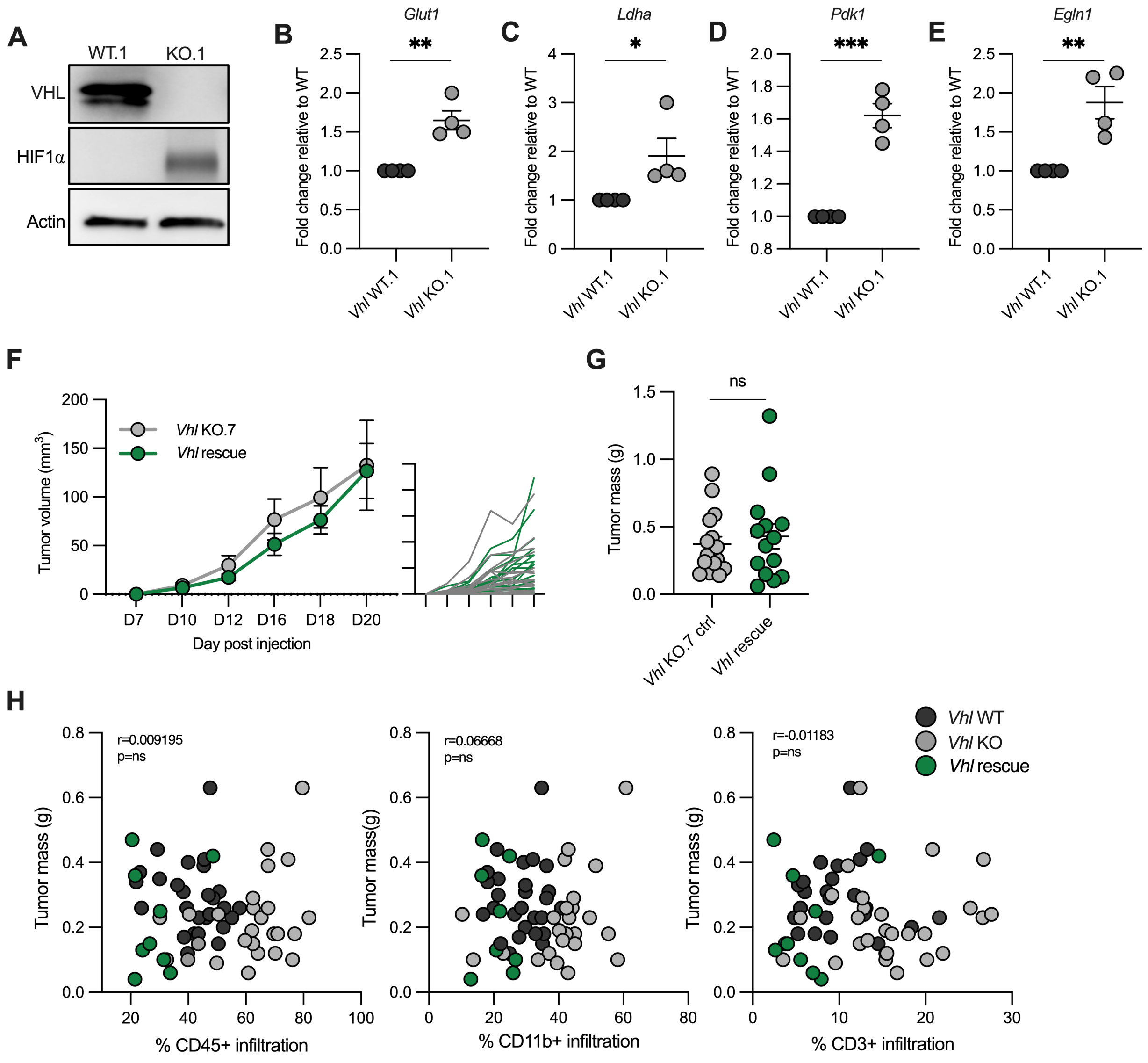

Supplementary Figure 2. Supporting data for figures 2 & 3.

(**A**) Representative western blot showing protein expression of VHL, HIF-1α, and Actin in Renca *Vhl* WT.2 or KO.1 cell lines. Quantitative PCR of (**B**) *Glut1,* (**C**) *Ldha,* (**D**) *Pdk1,* and (**E**), *Egln1,* in *Vhl* KO.1 cells relative to *Vhl* WT.2 cells. Each data point is a technical replicate from two independent experiments. (**F**) Average growth curve of *Vhl* KO.7 (grey) and *Vhl* Rescue (green) tumors represented as tumor volume (mm^3). Small graph shows biological replicates: *Vhl* KO.7 ctrl (n=14) and *Vhl* rescue (n=16)). (**G**) Final mass of each tumor from (**F**). (**H**) Dot plots of tumor mass versus $CD45^+$, $CD11b^+$, or $CD3^+$ immune cell infiltration in tumors formed from injection of *Vhl* WT, *Vhl* KO (including KO.1, KO.7 and KO.32 clones), and *Vhl* rescue cells. Each data point represents a biological replicate and graphs show mean and SEM. *P* values calculated using an unpaired, two-tailed Student's t-test (* $p<0.05$. ** $p<0.01$, *** $p<0.001$).

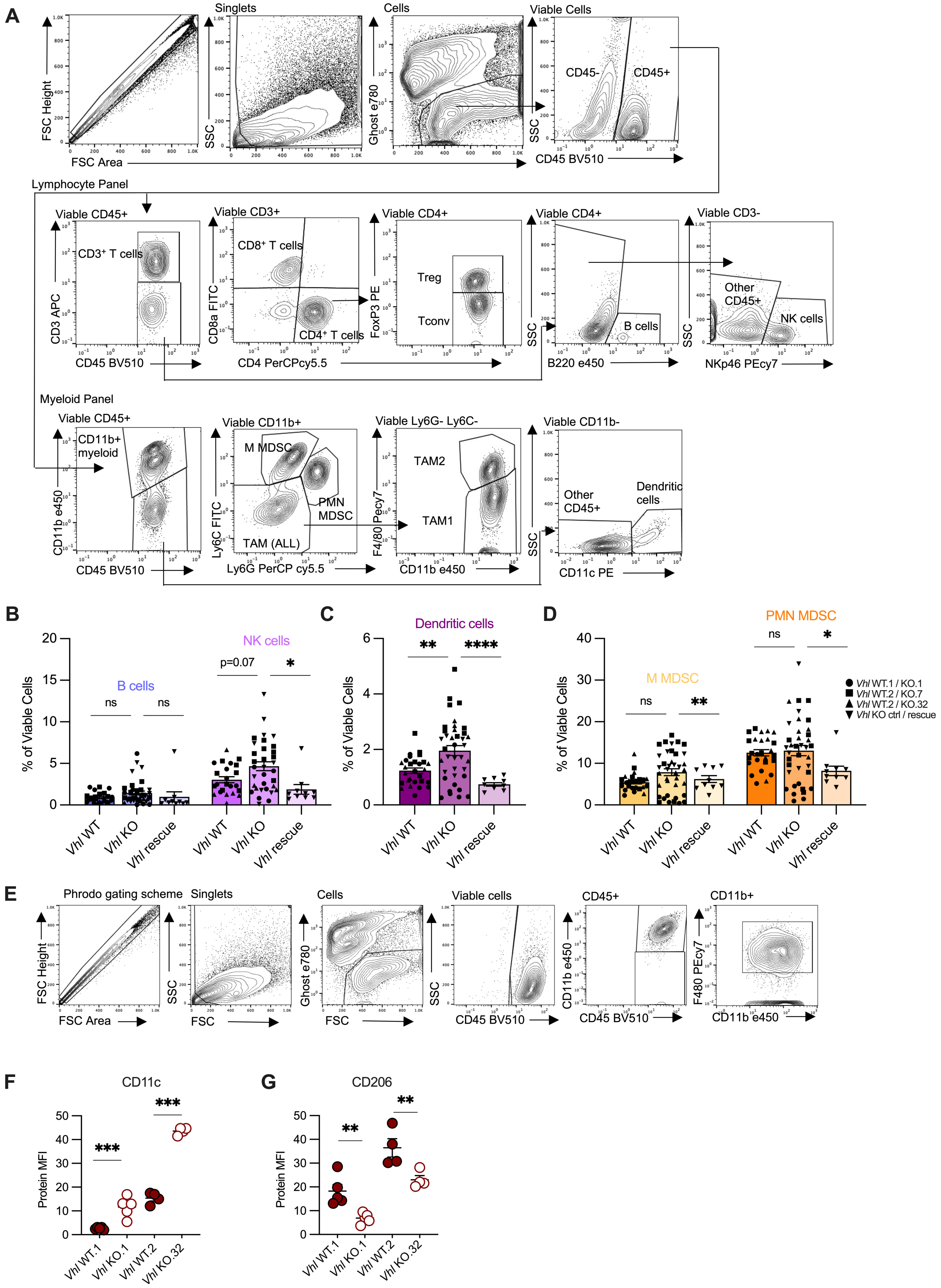
A
Singlets
Cells
Viable Cells
FSC Height
FSC Area
SSC
Ghost e780
CD45-
CD45+
CD45 BV510
Lymphocyte Panel
Viable CD45+
CD3⁺ T cells
CD3 APC
CD45 BV510
Viable CD3+
CD8⁺ T cells
CD4⁺ T cells
CD8a FITC
CD4 PerCPcy5.5
Viable CD4+
Treg
Tconv
FoxP3 PE
Viable CD4+
B cells
B220 e450
Viable CD3-
Other CD45+
NK cells
NKp46 PEcy7
Myeloid Panel
Viable CD45+
CD11b+ myeloid
CD11b e450
CD45 BV510
Viable CD11b+
M MDSC
PMN MDSC
TAM (ALL)
Ly6C FITC
Ly6G PerCP cy5.5
Viable Ly6G- Ly6C-
TAM2
TAM1
F4/80 Pecy7
CD11b e450
Viable CD11b-
Other CD45+
Dendritic cells
CD11c PE
B
B cells
NK cells
% of Viable Cells
ns
ns
p=0.07
*
Vhl WT
Vhl KO
Vhl rescue
C
Dendritic cells
**
****
D
M MDSC
PMN MDSC
ns
**
ns
*
Vhl WT.1 / KO.1
Vhl WT.2 / KO.7
Vhl WT.2 / KO.32
Vhl KO ctrl / rescue
E
Phrodo gating scheme
Singlets
Cells
Viable cells
CD45+
CD11b+
FSC Height
FSC Area
SSC
FSC
Ghost e780
CD45 BV510
CD11b e450
F480 PEcy7
F
CD11c
***
***
Protein MFI
Vhl WT.1
Vhl KO.1
Vhl WT.2
Vhl KO.32
G
CD206
**
**

Supplementary Figure 3. Renca tumor immune microenvironment characterization.

(**A**) Flow cytometry gating strategy for immune cell characterization using lymphocyte and myeloid-focused antibody panels. (DC: dendritic cell; M-MDSC: monocytic myeloid-derived suppressor cell; NK cell: natural killer cell; PMN-MDSC: polymorphonuclear myeloid-derived suppressor cell; TAM: tumor-associated macrophage). (**B-D**) Quantification of: B cells ($CD3^- B220^+$) and NK cells ($CD3^- B220^- Nkp46^+$), M-MDSC ($CD11b^+ Ly6G^- Ly6C^+$), PMN-MDSC ($CD11b^+ Ly6G^+ Ly6C^-$), and dendritic cells ($CD11b^- CD11c^+$), represented as % of all viable cells in each *Vhl* WT and *Vhl* KO tumor (experimental pairs represented by matched symbols). (**E**) Gating strategy for % $Phrodo^+$ cells from microbead-isolated $CD11b^+$ fractions. (**F** and **G**) Protein MFI quantification of CD11c and CD206 in overall TAM from *Vhl* WT.1/ KO.1 and *Vhl* WT.2/KO.32 paired tumors. Each data point represents a biological replicate and graphs show mean and SEM. *P* values determined by one-way ANOVA and Bonferroni's multiple comparison (**B-D**), two-tailed Student's t-test (**F** and **G**) (* p<0.05. ** p<0.01, ****p<0.0001).

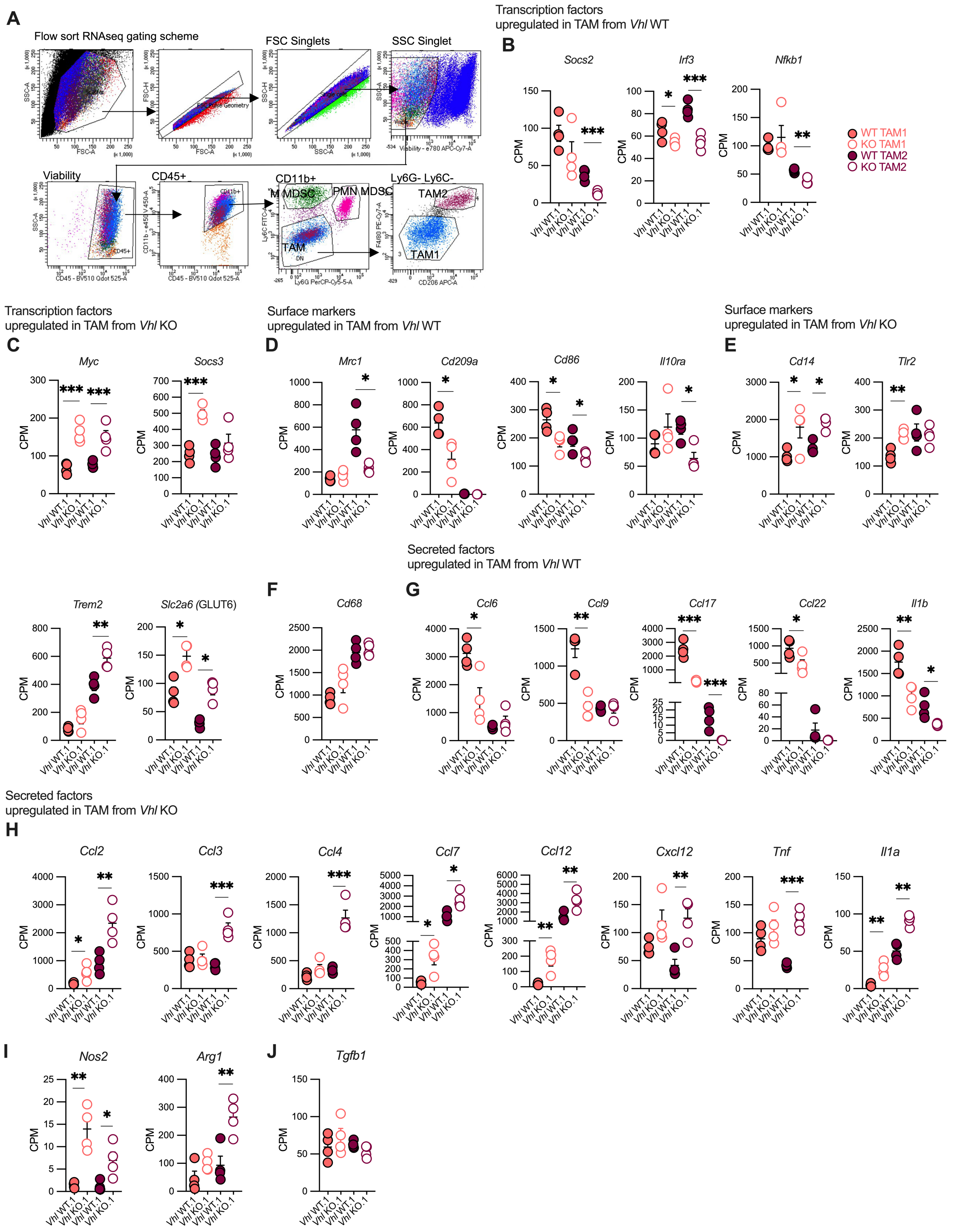

A
Flow sort RNAseq gating scheme
FSC Singlets
SSC Singlet
Viability
CD45+
CD11b+
M MDSC
PMN MDSC
Ly6G- Ly6C-
TAM2
TAM
TAM1
Transcription factors upregulated in TAM from Vhl WT
B
Socs2
Irf3
Nfkb1
WT TAM1
KO TAM1
WT TAM2
KO TAM2
Transcription factors upregulated in TAM from Vhl KO
C
Myc
Socs3
Surface markers upregulated in TAM from Vhl WT
D
Mrc1
Cd209a
Cd86
Il10ra
Surface markers upregulated in TAM from Vhl KO
E
Cd14
Tlr2
Trem2
Slc2a6 (GLUT6)
F
Cd68
Secreted factors upregulated in TAM from Vhl WT
G
Ccl6
Ccl9
Ccl17
Ccl22
Il1b
Secreted factors upregulated in TAM from Vhl KO
H
Ccl2
Ccl3
Ccl4
Ccl7
Ccl12
Cxcl12
Tnf
Il1a
I
Nos2
Arg1
J
Tgfb1
CPM
Vhl WT.1
Vhl KO.1

Supplementary Figure 4. *Vhl* loss promotes proinflammatory TAM transcriptional signatures.

(**A**) Gating strategy for flow sorted M MDSC, PMN MDSC, TAM1, and TAM2 myeloid populations from bead isolated CD11b$^+$ fractions. (**B**) Transcript count per million reads (CPM) of regulatory genes significantly upregulated in TAM populations from *Vhl* WT.1 tumors, and (**C**)**,** those upregulated in TAM from *Vhl* KO.1 tumors. (**D**) Transcript levels of surface marker genes significantly upregulated in TAM from *Vhl* WT.1 tumors, and (**E**)**,** *Vhl* KO.1 tumors. (**F**) *Cd68* TAM populations from WT.1 and KO.1. (**G**) Transcript counts of genes encoding secreted factors significantly upregulated in TAM populations from *Vhl* WT.2 tumors, and (**H**)**,** *Vhl* KO.1 tumors. (**I**) *Nos2* and *Arg1,* and (**J**)**,** *Tgfb* transcript CPM in TAM populations from WT.1 and KO.1. Each data point represents a biological replicate and graphs show mean and SEM. *P* values calculated using unpaired, two-tailed Student's t-test (* p<0.05. ** p<0.01, *** p<0.001, ****p<0.0001).

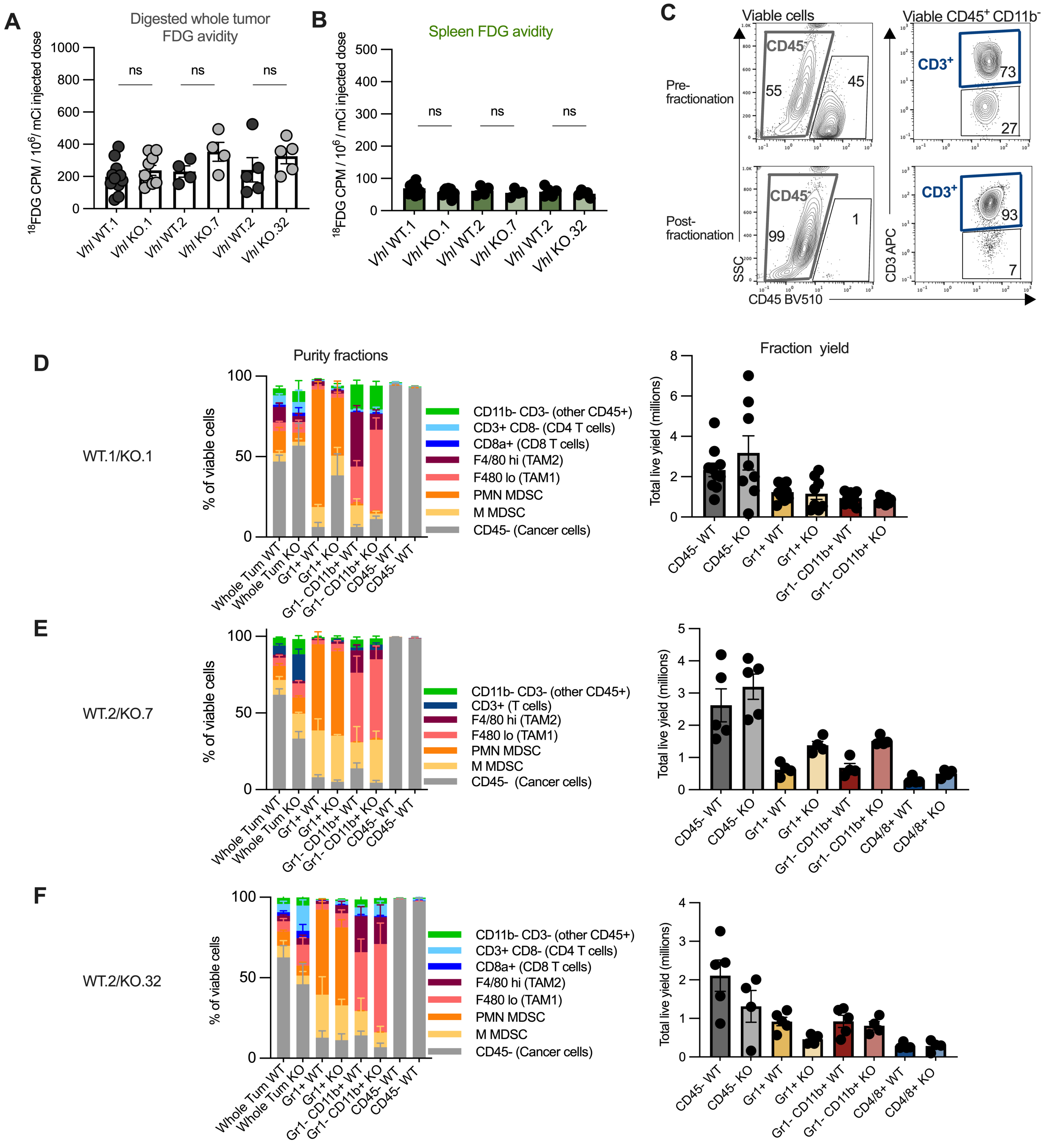

Supplementary Figure 5. Supportive data for Figure 6.

(**A**) Cellular FDG avidity of digested whole tumor single cell suspensions, and (**B**), splenic cells, in *Vhl* WT and matched designated *Vhl* KO clone. (**C**) Representative flow cytometry gating of whole tumor and CD45$^-$ and CD3$^+$ enriched fractions following

sequential isolation, and purity fractions and fraction yields of isolated Gr1^{+} (MDSC enriched), Gr1^{-} CD11b^{+} (TAM enriched), CD45^{-} (cancer cell enriched) fractions, and CD3^{+} (T cell enriched) fractions in (**D**), *Vhl* WT.1/KO.1, (**E**), *Vhl* WT.2/KO.7, and (**F**)**,** *Vhl* WT.2/KO.7. Each data point represents a biological replicate and graphs show mean and SEM.

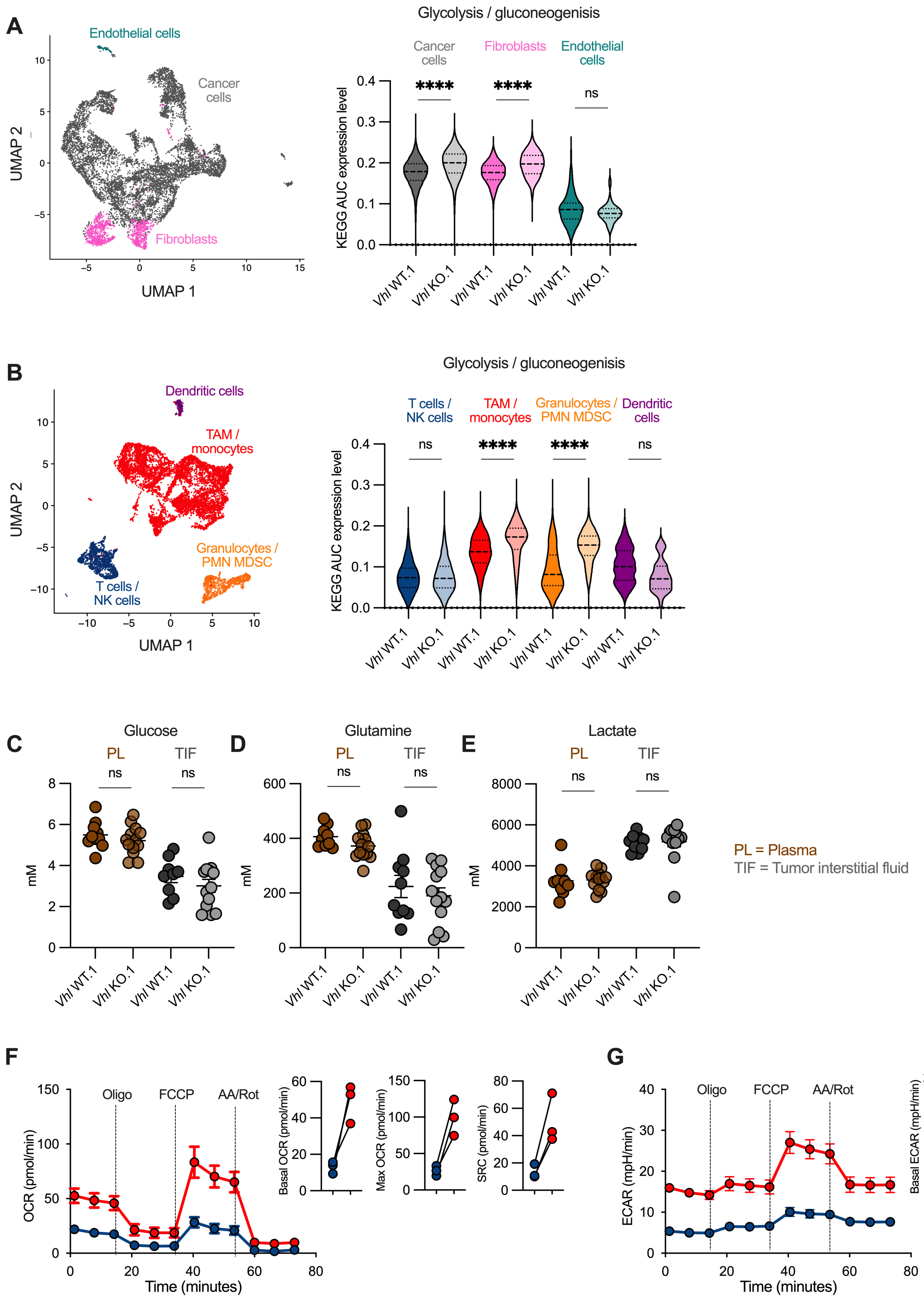
A
Endothelial cells
Cancer cells
Fibroblasts
UMAP 1
UMAP 2
Glycolysis / gluconeogenisis
Cancer cells
Fibroblasts
Endothelial cells
****
****
ns
KEGG AUC expression level
Vhl WT.1
Vhl KO.1
B
Dendritic cells
TAM / monocytes
Granulocytes / PMN MDSC
T cells / NK cells
UMAP 1
UMAP 2
Glycolysis / gluconeogenisis
T cells / NK cells
TAM / monocytes
Granulocytes / PMN MDSC
Dendritic cells
ns
****
****
ns
KEGG AUC expression level
C
Glucose
D
Glutamine
E
Lactate
PL
TIF
ns
mM
PL = Plasma
TIF = Tumor interstitial fluid
F
Oligo
FCCP
AA/Rot
OCR (pmol/min)
Time (minutes)
Basal OCR (pmol/min)
Max OCR (pmol/min)
SRC (pmol/min)
G
ECAR (mpH/min)
Basal ECAR (mpH/min)

Supplementary Figure 6. Single-cell transcriptome and metabolite analyses of Renca tumors.

(**A**) UMAP showing cell clusters in $CD45^-$ isolated cells subjected to single cell sequencing and KEGG pathway analyses for glycolysis/gluconeogenesis in designated non-immune cell populations. (**B**) UMAP showing cell clusters in $CD45^+$ isolated cells and KEGG pathway analyses for glycolysis/gluconeogenesis in designated immune cell populations. GC/MS quantification of (**C**) glucose, (**D**) glutamine, and (**E**) lactate from plasma and tumor interstitial fluid (TIF). Seahorse flux analysis of (**F**) OCR and (**G**), ECAR from mitochondrial stress test in microbead isolated $CD11b^+$ and $CD3^+$ cells from n=3 ccRCC patient tumors. *P* values calculated using Wilcoxon matched pairs signed rank test (**A** and **B**). *P* values calculated using unpaired, two-tailed Student's t-test (**C-E**) (****p<0.0001).

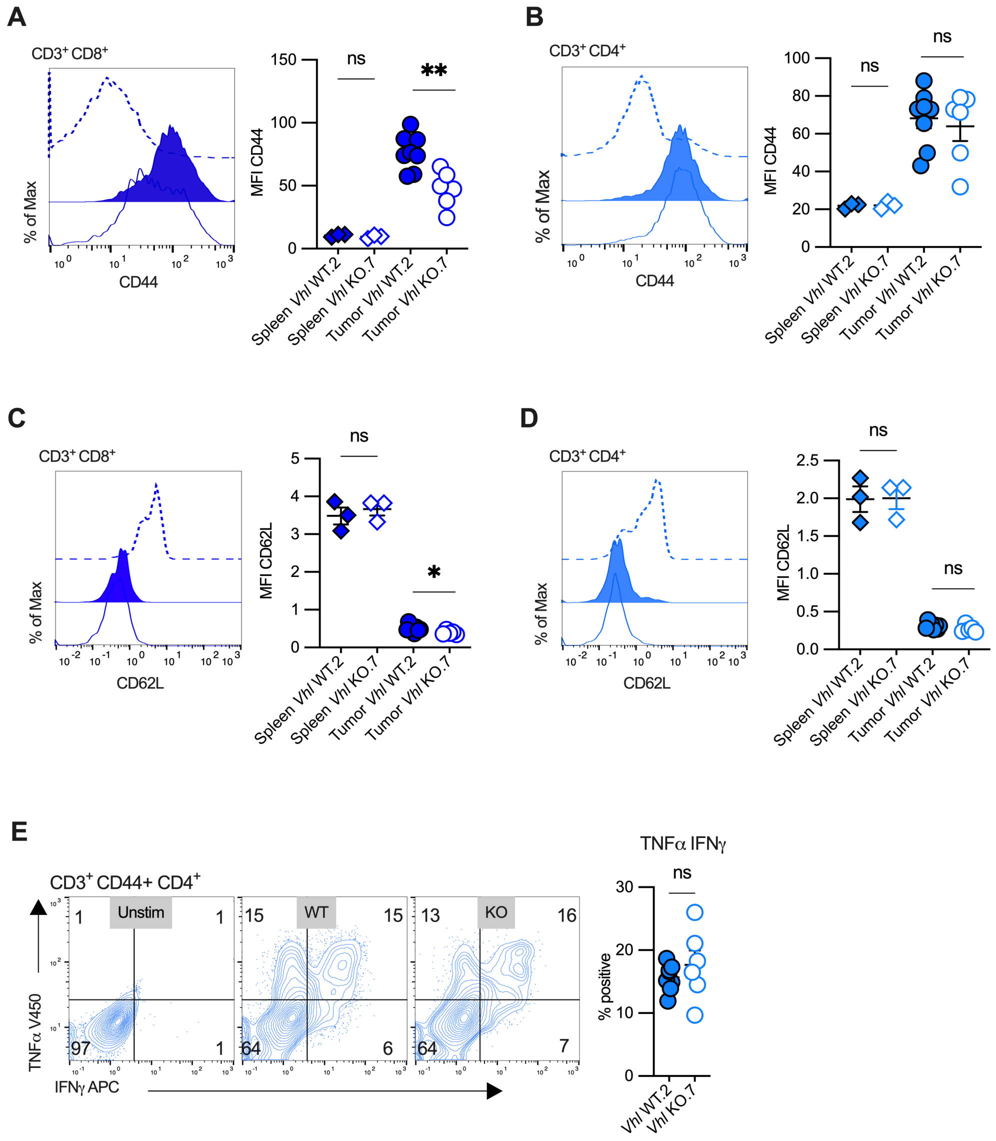

Supplementary Figure 7. T cells residing in the *Vhl* KO TME are dysfunctional. Representative histograms and MFI quantification of CD44 in (**A**), CD3$^+$ CD8$^+$ T cells, and (**B**), CD3$^+$ CD4$^+$ T cells, and CD62L, in (**C**) CD3$^+$ CD8$^+$, and (**D**), CD3$^+$ CD4$^+$ T cells from spleen or tumor in mice harboring genetically distinct tumors. (**E**) Representative flow plots and quantification of TNFα IFNγ producing CD3$^+$ CD44$^+$ CD4$^+$ cells from *Vhl* WT and KO tumors. Each data point represents a biological replicate and graphs show

mean and SEM. *P* values calculated using two-tailed Student's T test. (* p<0.05. ** p<0.01, ****p<0.0001).

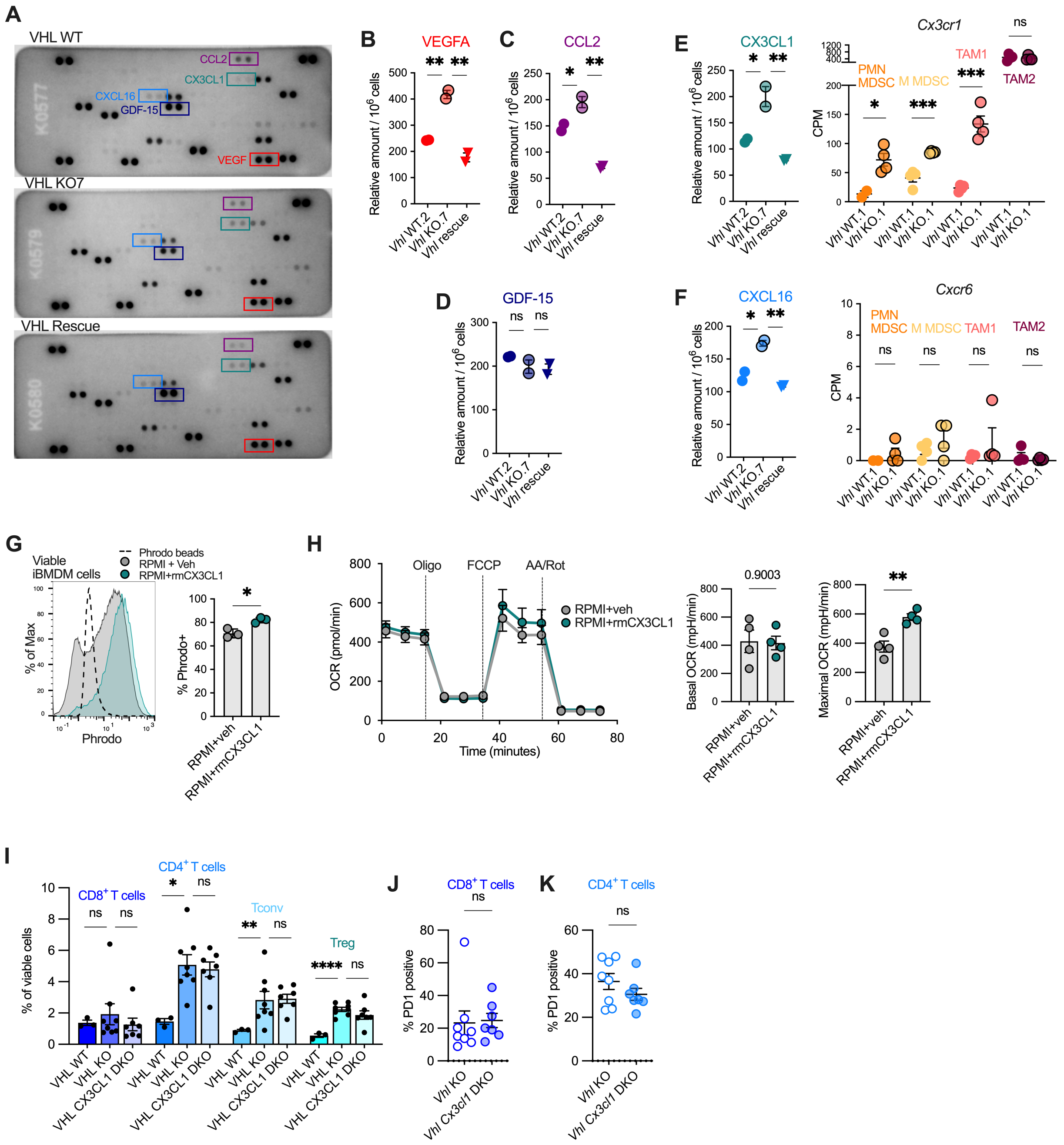

Supplementary Figure 8. Secreted factors from *Vhl* deficient Renca cells.

(**A**) Cytokine microarray from conditioned media in *Vhl* WT.1, KO.7 or rescue clones. Quantification of (**B**) VEGFA, (**C**) CCL2, and (**D**) GDF-15 from indicated clone

normalized to cell number. (**E**) Quantification of secreted CX3CL1 and *Cx3cr1* transcript counts per million (CPM) from flow sorted bulk RNA sequencing of indicated populations from Renca tumors with indicated *Vhl* status. (**F**) Quantification of secreted CXCL16 in supernatant and *Cxcr6* transcript reads from indicated myeloid population. (**G**) Histogram representing Phrodo$^+$ uptake in immortalized bone marrow derived macrophages (iBMDM) treated with either RPMI + vehicle or RPMI + recombinant mouse (rmCX3CL1). Seahorse flux analysis of (**H**) OCR of mitochondrial stress test in iBMDM treated with either vehicle or rmCX3CL1. (**I**) Quantification of specified lymphocyte populations as % of viable cells in *Vhl* WT, *Vhl* KO or *Vhl Cx3cl1* DKO tumors. See Supplemental Figure 3 for complete gating strategy. (**J**) % PD1 positive CD8$^+$ T cells, and (**K**) CD4$^+$ T cells in *Vhl* KO and *Vhl Cx3cl1* DKO tumors. Data points represent technical replicates and graphs show mean and SEM (**B**, **C**, **D**, **E** (CX3CL1), and **F** (CXCL16)), and biological replicates (**E** *(Cx3cr1)* **F** *(Cxcr6),* and **J**-**K**). *P* values calculated using unpaired, two-tailed Student's t-test (* $p<0.05$. ** $p<0.01$, *** $p<0.001$, ****$p<0.0001$).
