# Supplementary figures and images for: VHL loss reprograms the immune landscape to promote an inflammatory myeloid microenvironment in renal tumorigenesis
Source: J Clin Invest. 2024 Apr 15;134(8):e173934. doi: 10.1172/JCI173934 (PMC11014672; doi:10.1172/JCI173934)

# Original unedited western blots

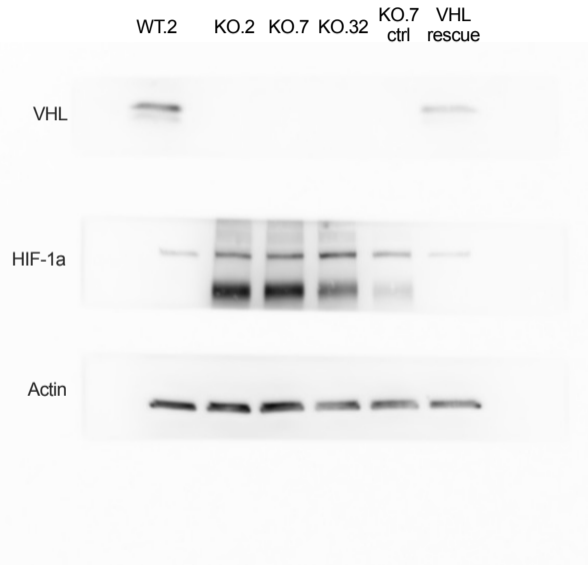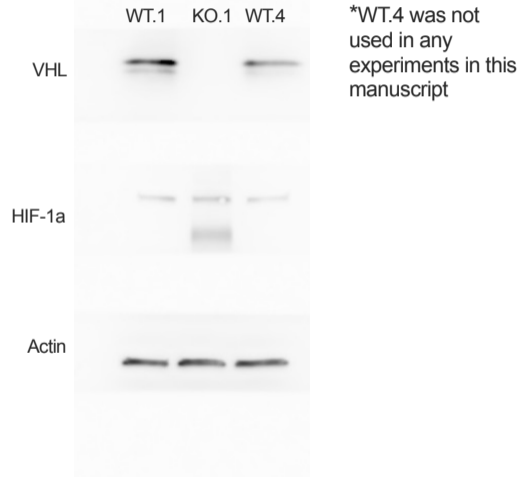

Supplement: Unedited blot and gel images [file jci-134-173934-s137.pdf]
